# Low Versus High Intraoperative Intraocular Pressure During Phacoemulsification Cataract Surgery: A Systematic Review and Meta-Analysis of Randomized Controlled Trials

**DOI:** 10.3390/jcm15218557

**Published:** 2026-11-03

**Authors:** Ahmed Abu-Zaid, Omar A. Alnajar, Rashed A. Alasoosi, Fawaz Almutairi, Abdullah M. Alazemi, Latifah Alyaqout, Mariam Mohammad Alkandari, Farah R. Alqussair, Abdulaziz S. Alasfar, Omar Almutairi, Maryam S. AlAmrani, Abdullah J. Alrashed, Abdullah M. Alharran

**Affiliations:** 1 Department of Biochemistry and Molecular Medicine, College of Medicine, Alfaisal University, Riyadh 11533, Saudi Arabia; 2 Ministry of Health, Kuwait City 13001, Kuwait; 3 Kuwait Institute for Medical Specializations, Kuwait City 12050, Kuwait; 4 Faculty of Medicine, Jordan University of Science and Technology, Irbid 22110, Jordan; 5 College of Medicine and Medical Sciences, Arabian Gulf University, Manama 26671, Bahrain

**Keywords:** active fluidics, active sentry, cataract surgery, intraoperative intraocular pressure, low IOP, meta-analysis, phacoemulsification

## Abstract

**Objective:** To conduct a systematic review and meta-analysis of randomized controlled trials (RCTs) comparing low versus high intraoperative intraocular pressure (IOP) during phacoemulsification. **Methods:** PubMed, Scopus, Web of Science (WoS), and CENTRAL were searched from inception through 6 July 2026, followed by a supplementary manual search conducted before data extraction on 5 August 2026, to identify any additional eligible studies. RCTs comparing low-IOP versus high-IOP strategies during phacoemulsification were included. Outcomes were categorized as intraoperative, postoperative, and safety outcomes. Data were pooled as mean differences (MDs) or risk ratios (RRs) with corresponding 95% confidence intervals (CIs) using random-effects models. **Results:** Twelve RCTs comprising 1051 eyes were included. Cumulative dissipated energy was comparable between low- and high-IOP strategies (MD −0.12, 95% CI −0.45 to 0.20; *p* = 0.42). No significant between-group differences were observed in endothelial cell density, best-corrected visual acuity, central corneal thickness, or postoperative IOP across the reported follow-up periods (all *p* > 0.05). Intraoperative pain, aspiration time, total case time, fluid usage, and safety outcomes were also comparable between groups. Low-IOP phacoemulsification was associated with a modest increase in total ultrasound time (MD 1.53 s, 95% CI 0.03 to 3.03; *p* = 0.047). **Conclusions:** Low-IOP phacoemulsification provides intraoperative, postoperative, and safety outcomes broadly comparable to high-IOP strategies. Despite a statistically significant but clinically negligible increase in ultrasound time of approximately 1.5 s in favor of low-IOP, no significant differences were observed in cumulative dissipated energy, endothelial cell density, best-corrected visual acuity, central corneal thickness, postoperative IOP, or safety outcomes. However, these findings should not be interpreted as definitive evidence of equivalence or non-inferiority between the two strategies.

## 1. Introduction

Cataract remains one of the leading causes of visual impairment and blindness worldwide [1,2], and phacoemulsification with intraocular lens implantation is the standard surgical treatment [3,4]. Although modern phacoemulsification is highly effective and safe, maintaining a stable anterior chamber throughout the procedure remains essential for surgical efficiency and optimal postoperative outcomes [5].

Intraoperative intraocular pressure (IOP) is largely determined by the balance between irrigation and aspiration [6]. Conventional gravity-based fluidic systems typically require elevated irrigation bottle heights to maintain chamber stability and reduce post-occlusion surge [7]. This often results in IOP levels that exceed the physiological range [8]. While higher IOP may facilitate chamber maintenance during surgery, concerns have been raised regarding its potential effects on ocular tissues, including endothelial cell damage, corneal edema, reduced ocular perfusion, and patient discomfort [9].

Recent advances in fluidics technology have enabled phacoemulsification to be performed at lower, more physiological IOP levels while maintaining adequate chamber stability [10]. Active-fluidics systems continuously regulate fluid delivery and compensate for fluctuations within the anterior chamber, allowing more precise control of IOP during surgery [11]. These developments have generated increasing interest in whether low-IOP settings can improve surgical and postoperative outcomes without compromising procedural safety or efficiency [12].

Several randomized controlled trials (RCTs) have compared low intraoperative IOP with higher-pressure approaches during phacoemulsification [10,13]. Although these studies have evaluated a range of intraoperative and postoperative outcomes, their findings have been inconsistent.

To date, no systematic review or meta-analysis has specifically examined the efficacy and safety of low versus high intraoperative IOP during phacoemulsification, leaving the overall effect of this strategy uncertain. Therefore, we conducted a systematic review and meta-analysis of RCTs to evaluate the efficacy and safety of low versus high intraoperative IOP during phacoemulsification cataract surgery.

## 2. Methods and Materials

### 2.1. Study Design and Registration

This systematic review and meta-analysis was conducted in accordance with the Cochrane Handbook for Systematic Reviews of Interventions [14] and reported following the Preferred Reporting Items for Systematic Reviews and Meta-Analyses (PRISMA) 2020 statement [15]. The study protocol was prospectively registered in the International Prospective Register of Systematic Reviews (PROSPERO; CRD420261496866). Table S1 shows the PRISMA checklist.

### 2.2. Literature Search and Study Selection

A comprehensive literature search was performed in PubMed, Scopus, Web of Science (WoS), and the Cochrane Central Register of Controlled Trials (CENTRAL) from database inception through 6 July 2026, followed by a supplementary manual search conducted before data extraction on 5 August 2026, to identify any additional eligible studies. The search strategy combined terms related to cataract surgery, phacoemulsification, and intraoperative intraocular pressure, including the keywords: cataract surgery, phacoemulsification, fluidics, and intraocular pressure. No language or publication-type restrictions were applied during the initial database searches. Non-English publications were subsequently excluded during eligibility assessment according to the predefined inclusion criteria. The complete search strategies for all databases are provided in the Table S2.

### 2.3. Study Selection Process

All retrieved records were imported into EndNote for duplicate removal and subsequently uploaded to Rayyan for screening. Two reviewers independently screened titles and abstracts for potential eligibility. Full-text articles of potentially relevant studies were then assessed against the predefined eligibility criteria. Disagreements were resolved through discussion and consensus. In addition, forward and backward citation searches were performed manually to identify any additional eligible studies.

### 2.4. Eligibility Criteria and Outcomes

We included RCTs meeting the following PICO criteria: population (P): patients undergoing phacoemulsification cataract surgery; intervention (I): low-IOP settings (target intraoperative pressures approximating the physiological range at 20–30 mmHg), comparator (C): high-IOP settings (target higher intraoperative pressure settings approximately at 50–80 mmHg or equivalent bottle-height settings) [16,17], outcomes (O): grouped into intraoperative, postoperative, and safety outcomes. Intraoperative outcomes included cumulative dissipated energy (CDE; CDE units), intraoperative pain (visual analog scale [VAS] score), aspiration time (seconds), total ultrasound time (seconds), total case time (seconds), irrigation fluid usage (mL), and aspirated fluid volume (mL). Postoperative outcomes included changes in corneal endothelial cell density (cells/mm^2^), best-corrected visual acuity (BCVA; logMAR), central corneal thickness (CCT; µm), and intraocular pressure (IOP; mmHg). Safety outcomes included postoperative corneal edema, posterior capsule rupture, and clinically defined postoperative IOP elevation.

We further excluded observational studies, non-randomized trials, review articles, editorials, letters, conference abstracts lacking sufficient data for extraction, and non-English publications.

### 2.5. Data Extraction and Risk of Bias Assessment

Data were extracted independently by two reviewers using a standardized, pilot-tested electronic data extraction form. Extracted information included study-level characteristics (study design, country, recruitment period, sample size, fluidics platform, surgical technique, follow-up duration, and study conclusions), patient-level characteristics (age, sex, eye laterality, anterior chamber depth, central corneal thickness, endothelial cell density, and axial length), risk of bias assessments, and all predefined efficacy and safety outcomes. Any discrepancies in data extraction were resolved through discussion and consensus among the investigators.

The methodological quality of included RCTs was assessed using the Cochrane Risk of Bias 2 (ROB-2) tool [18]. Five domains were evaluated: bias arising from the randomization process, deviations from intended interventions, missing outcome data, outcome measurement, and selection of the reported result. Each domain was rated as low risk of bias, some concerns, or high risk of bias, leading to an overall risk of bias judgment for each study.

### 2.6. Statistical Analysis

For continuous outcomes, pooled effect estimates were calculated as mean differences (MDs) with corresponding 95% confidence intervals (CIs). For endothelial cell density, the analysis used changes from baseline to the reported postoperative time points rather than absolute postoperative measurements, where change-score data were available. For dichotomous outcomes, pooled risk ratios (RRs) with 95% CIs were calculated. Meta-analyses were conducted using random-effects models with Hartung–Knapp adjustment to account for potential between-study variability. Statistical heterogeneity was assessed using Cochran’s Q test and quantified using I^2^. I^2^ values of approximately 50% or greater were considered indicative of at least moderate heterogeneity.

When sufficient data were available, postoperative outcomes reported at multiple timepoints were analyzed separately according to follow-up duration. For multi-arm trials containing more than one eligible low- versus high-IOP comparison with distinct participants, each independent comparison was entered separately. Specifically, for Vasavada et al. study [19], the Traditional and Active Sentry comparisons were included separately when outcome-specific data were available for both comparisons. When data were available for only one comparison, only that comparison was included in the corresponding meta-analysis. For paired-eye trials, eyes were treated as independent observations because within-patient correlation coefficients were not consistently reported. Consequently, paired-eye and parallel-group trials were pooled using the available aggregate data without adjustment for within-patient correlation. Leave-one-out sensitivity analysis was performed to evaluate the influence of individual comparisons on the pooled estimates whenever possible. A post hoc subgroup analysis was conducted for CDE based on whether the intervention and comparator groups used the same or different fluidics platforms or associated technologies.

Publication bias was assessed using funnel plots to visualize the association between effect size and standard error, and Egger’s regression test was performed to assess statistical evidence of small-study effects. A two-sided *p*-value < 0.05 was considered statistically significant for hypothesis testing, whereas a *p*-value < 0.10 in Egger’s regression test was considered indicative of potential small-study effects. All statistical analyses were performed using R software version 4.2 (R Foundation for Statistical Computing, Vienna, Austria).

## 3. Results

### 3.1. Search Results

**Figure 1:**
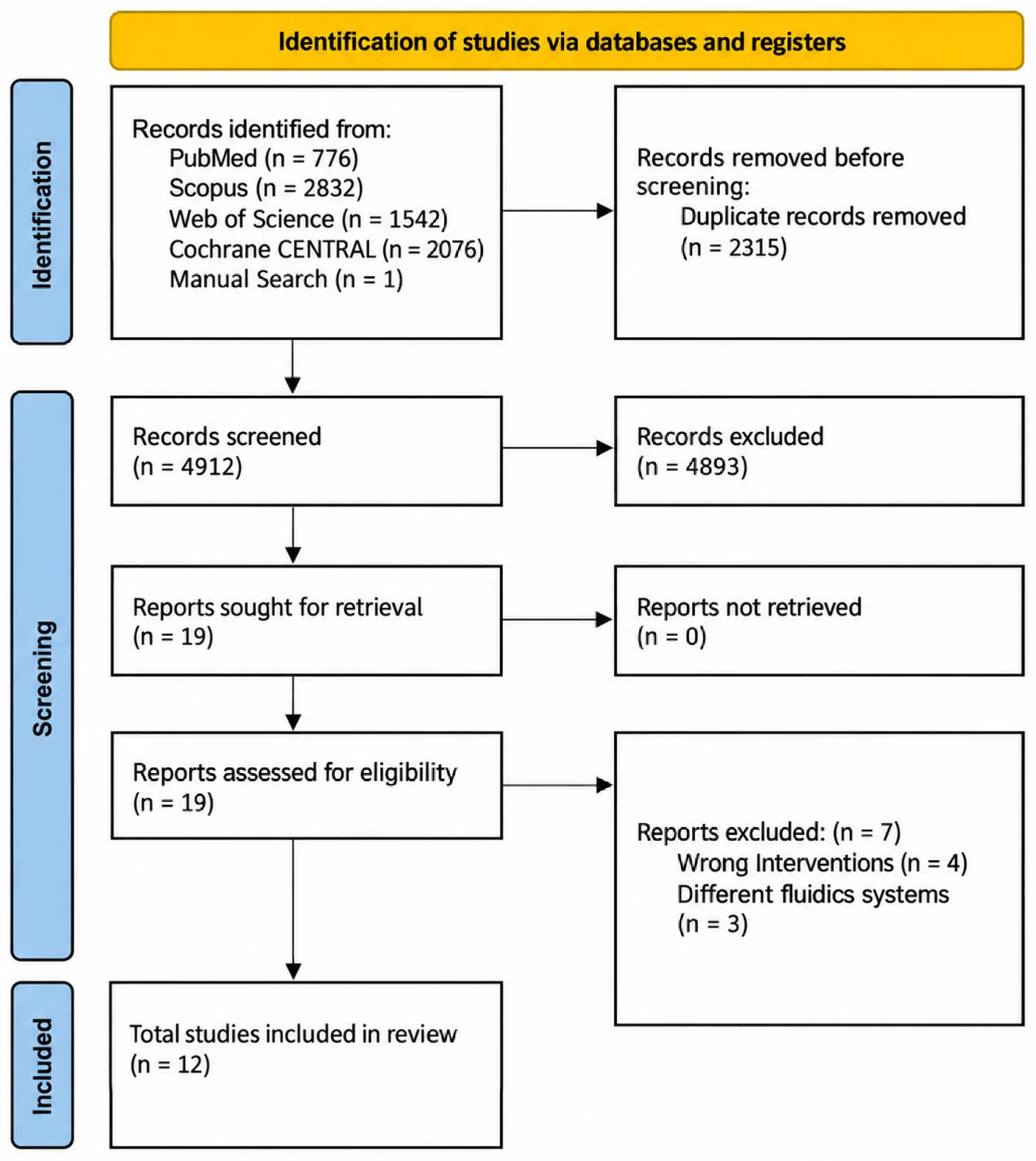
Preferred Reporting Items for Systematic Reviews and Meta-Analyses (PRISMA) flow diagram of the study selection process. The flow diagram summarizes the identification, screening, eligibility assessment, and final inclusion of studies in the systematic review and meta-analysis.

The literature search identified 7267 records. After removal of 2315 duplicates, 4951 records underwent title and abstract screening, of which 4932 were excluded. The full texts of 19 reports were assessed for eligibility, of which seven were excluded, resulting in the inclusion of 12 RCTs comprising 1051 eyes [10,13,16,19,20,21,22,23,24,25,26,27]. A list of potentially relevant full-text studies excluded after eligibility assessment is provided in Table S3. The study selection process is summarized in the PRISMA flow diagram (Figure 1).

### 3.2. Characteristics of Included Studies and Participants

The 12 included RCTs were published between 2023 and 2026 and were conducted across Austria, China, the United Kingdom, the United States, Japan, Argentina, and India. Overall, the included trials comprised 1051 eyes, of which 526 were assigned to low-IOP phacoemulsification and 525 to high-IOP phacoemulsification. All studies compared low-IOP and high-IOP phacoemulsification strategies, although several trials also differed with respect to fluidics systems, phacoemulsification platforms, or handpiece technology. Target low-IOP settings generally ranged between 20 and 30 mmHg, whereas high-IOP settings ranged from approximately 50 to 65 mmHg or were achieved using equivalent bottle-height settings. Mean participant age ranged from 64.8 to 75.8 years. Baseline ocular characteristics were generally balanced between study groups, although some variability in baseline endothelial cell density was observed across studies. Detailed study and patient characteristics are presented in Table 1 and Table 2.

### 3.3. Risk of Bias Assessment Summary

**Figure 2:**
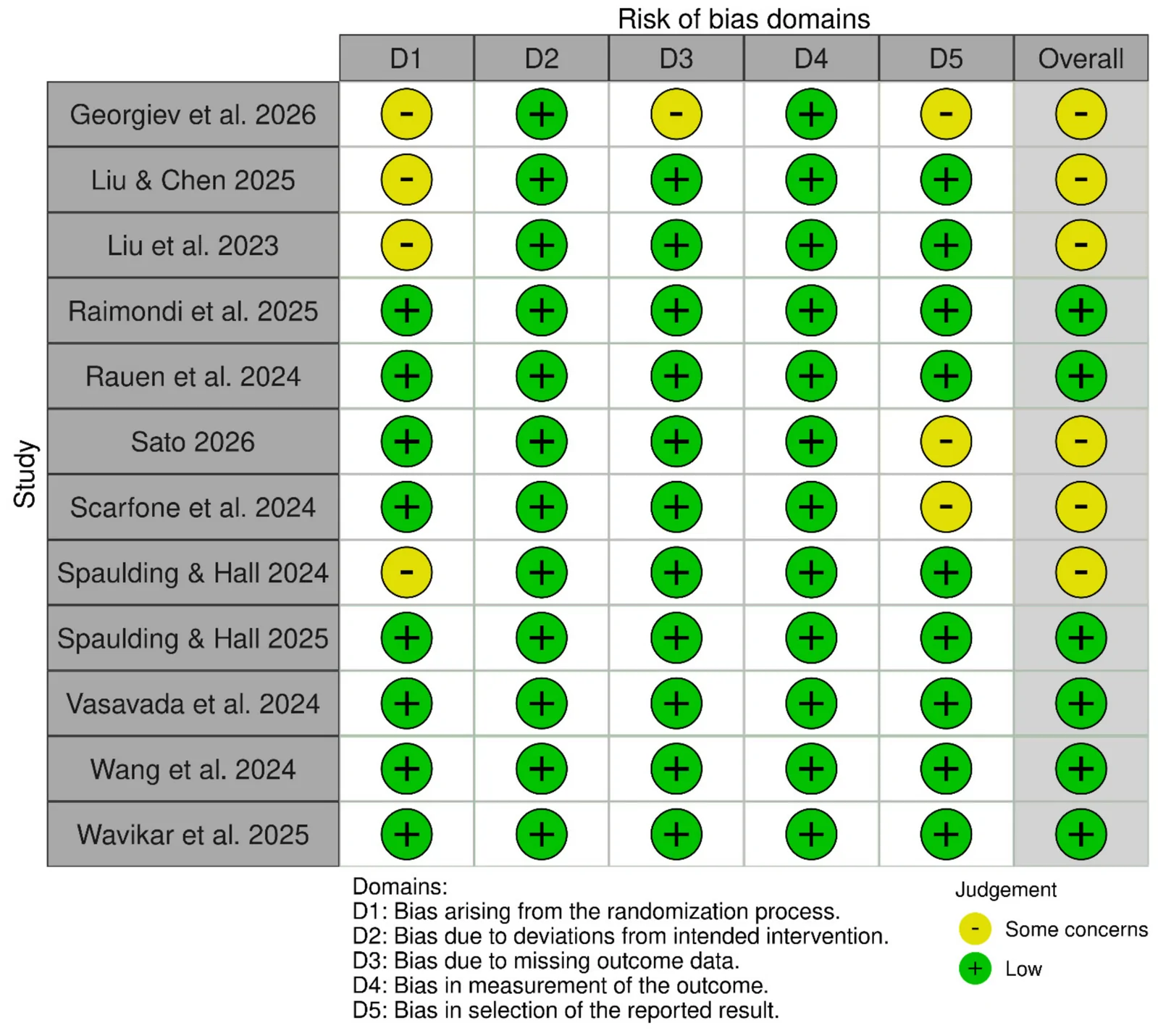
Risk of bias assessment summary of the included randomized controlled trials. Risk of bias was evaluated using the Cochrane Risk of Bias 2 (RoB-2) tool across five domains [10,13,16,19,20,21,22,23,24,25,26,27].

Six trials were judged to have a low overall risk of bias, and six were judged as having some concerns; no trial was rated at high risk of bias. Some concerns related to the randomization process were identified for Georgiev et al. (2026) [27], Liu and Chen (2025) [13], Liu et al. (2023) [16], and Spaulding and Hall (2024) [21]. Georgiev et al. (2026) [27] also had some concerns regarding missing outcome data, while Georgiev et al. (2026) [27], Scarfone et al. (2024) [26], and Sato (2026) [10] had some concerns regarding selection of the reported result. All remaining domain judgments were low risk (Figure 2).

### 3.4. Intraoperative Outcomes

#### 3.4.1. Cumulative Dissipated Energy (Units)

**Figure 3:**
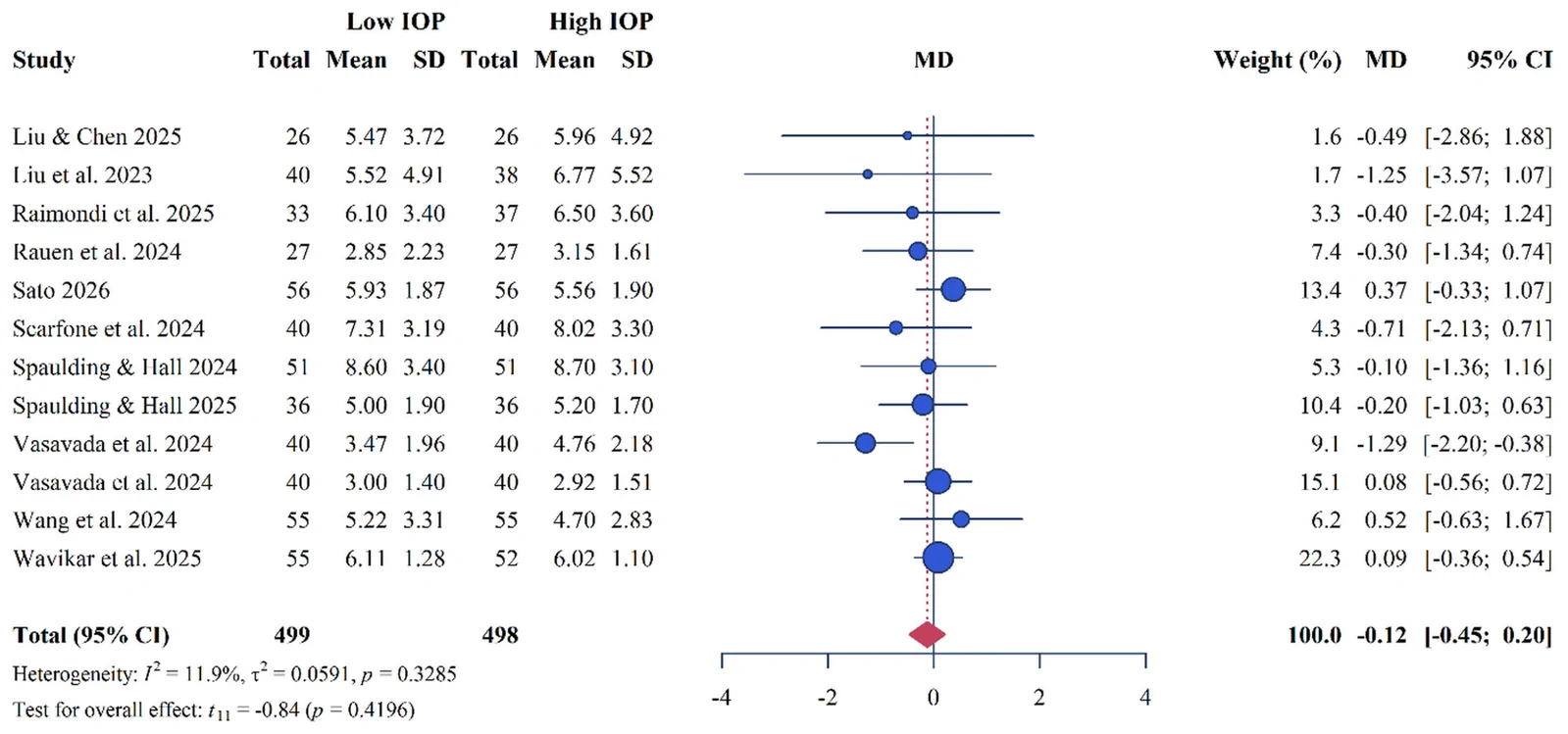
Forest plot of cumulative dissipated energy (CDE), presented as mean differences (MDs) with corresponding 95% confidence intervals (Cis). For Vasavada et al., 2024 [19], the Traditional and Active Sentry comparisons are presented separately, where both contributed eligible outcome data [10,13,16,19,20,21,22,23,24,25,26].

Eleven RCTs contributed 12 independent comparisons of CDE (one trial provided two eligible comparisons) including 499 eyes in the low-IOP group and 498 eyes in the high-IOP group. Low-IOP was not associated with a significant difference in CDE compared with high-IOP (MD −0.12 CDE units, 95% CI −0.45 to 0.20; *p* = 0.42), with low heterogeneity (I^2^ = 11.9%) (Figure 3). Leave-one-out sensitivity analysis did not materially alter the pooled estimate (Figure S1). Egger’s test did not indicate significant small-study effects (*p* = 0.136; Figure S2). Subgroup analysis of CDE according to the fluidics system comparison revealed no statistically significant differences between low and high intraocular pressure (IOP) settings. In studies comparing different fluidics systems (AFS vs. GFS), the pooled mean difference (MD) was −0.88 (95% CI: −5.71 to 3.95; *p* = 0.260; I^2^ = 0%). Similarly, studies comparing the same fluidics system (AFS vs. AFS) showed no significant difference (MD = −0.10; 95% CI: −0.46 to 0.26; *p* = 0.552; I^2^ = 20.8%). The test for subgroup differences was not statistically significant (*p* = 0.058), suggesting no conclusive evidence that the type of fluidics system comparison modified the effect of IOP settings on CDE (Figure S3).

#### 3.4.2. Intraoperative Pain (VAS)

Four studies reported intraoperative pain. No significant difference was observed between low- and high-IOP phacoemulsification (RR 0.28, 95% CI 0.03 to 2.44; *p* = 0.16), although substantial heterogeneity was present (I^2^ = 71.6%; Figure S4). Leave-one-out sensitivity analysis did not materially alter the pooled estimate (Figure S5).

#### 3.4.3. Operative Efficiency and Fluidics Parameters

Operative efficiency outcomes were largely comparable between groups. No significant differences were observed in aspiration time (MD 4.50 s, 95% CI −4.20 to 13.20; *p* = 0.22; I^2^ = 46.4%; Figure S6) or total case time (MD −3.38 s, 95% CI −32.65 to 25.89; *p* = 0.78; I^2^ = 49.9%; Figure S7). However, low-IOP was associated with a modest increase in total ultrasound time compared with high-IOP (MD 1.53 s, 95% CI 0.03 to 3.03; *p* = 0.047; I^2^ = 0%; Figure S8).

Fluidics-related outcomes were also similar between groups. Neither irrigation fluid usage (MD −4.04 mL, 95% CI −10.10 to 2.02; *p* = 0.16; I^2^ = 81.2%; Figure S9) nor aspirated fluid volume (MD −2.67 mL, 95% CI −12.21 to 6.87; *p* = 0.35; I^2^ = 68.0%; Figure S10) differed significantly between treatment strategies.

Leave-one-out sensitivity analyses did not materially alter the pooled estimates for most outcomes. For total ultrasound time, however, the pooled effect became statistically non-significant in all leave-one-out scenarios except when Spaulding and Hall 2025 [22] was excluded, in which case the pooled estimate remained statistically significant (Figures S11–S15).

### 3.5. Postoperative Outcomes

#### 3.5.1. Change in Endothelial Cell Density (Cells/mm^2^)

**Figure 4:**
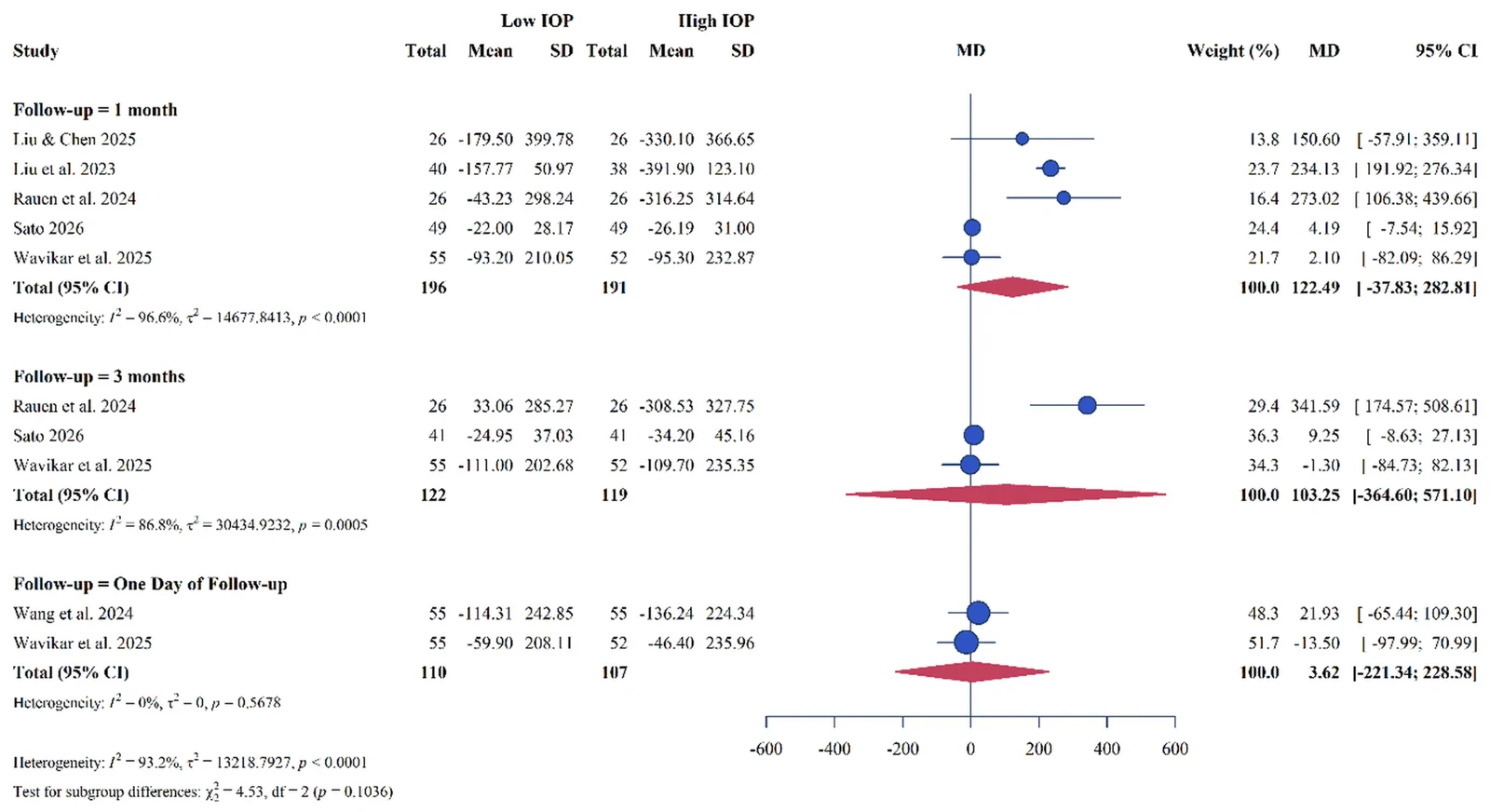
Forest plot of endothelial cell density (ECD), presented as mean differences (MDs) with corresponding 95% confidence intervals (CIs) [10,13,16,23,24,25].

Postoperative changes in endothelial cell density were analyzed separately by follow-up duration. No significant between-group differences were observed on postoperative day 1 (MD 3.62 cells/mm^2^, 95% CI −221.34 to 228.58; I^2^ = 0%), at 1 month (MD 122.49 cells/mm^2^, 95% CI −37.83 to 282.81; I^2^ = 96.6%), or at 3 months (MD 103.25 cells/mm^2^, 95% CI −364.60 to 571.10; I^2^ = 86.8%) (Figure 4). The test for subgroup differences according to follow-up duration was not significant (*p* = 0.103). Substantial heterogeneity was present in the 1-month and 3-month analyses. Egger’s test did not indicate significant small-study effects (*p* = 0.158; Figure S16).

#### 3.5.2. Best-Corrected Visual Acuity (logMAR)

**Figure 5:**
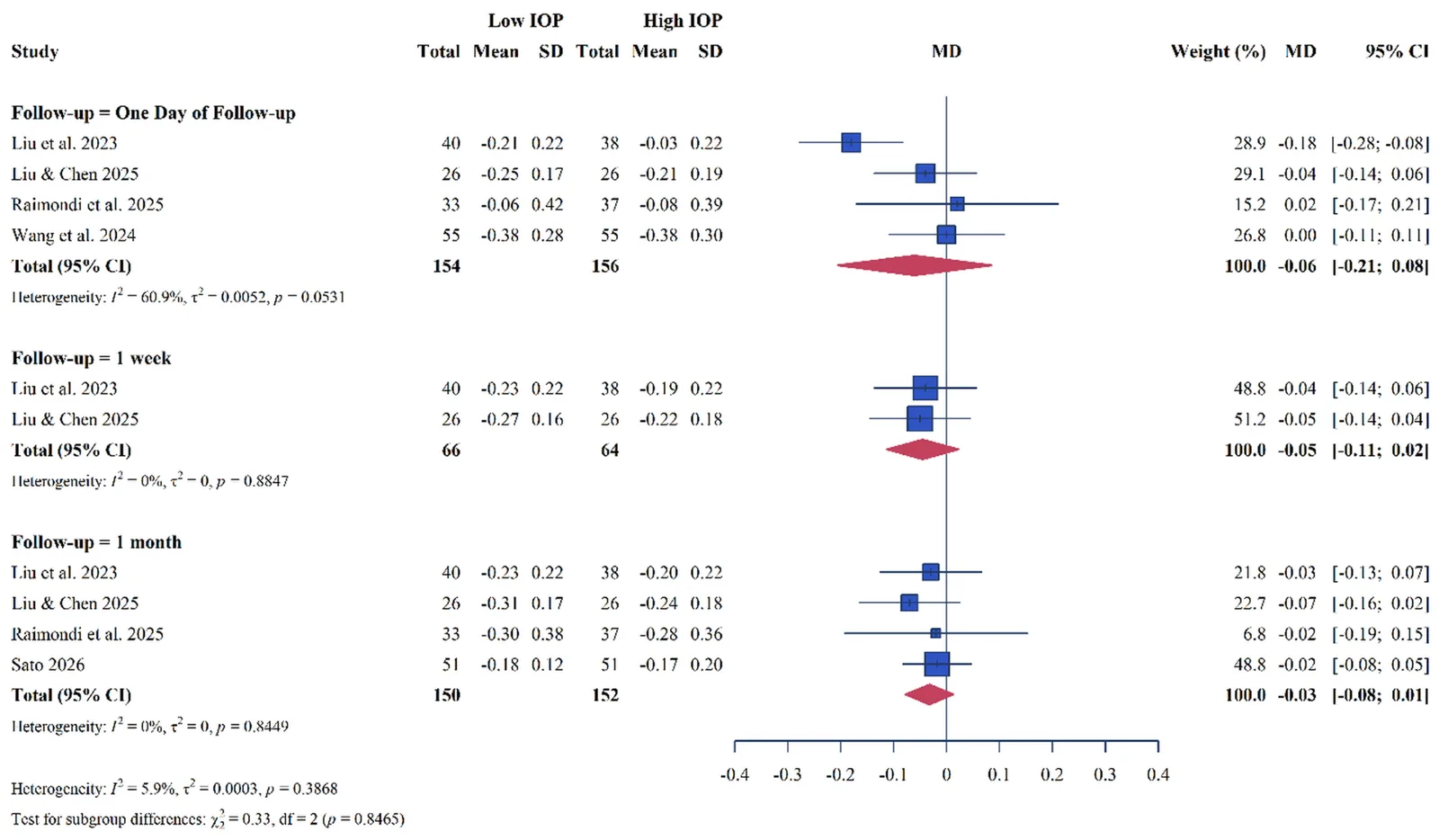
Forest plot of best-corrected visual acuity (BCVA), presented as mean differences (MDs) with corresponding 95% confidence intervals (CIs) [10,13,16,20,24].

No significant differences were observed in the change in BCVA between the low- and high-IOP groups on postoperative day 1 (MD −0.06 logMAR, 95% CI −0.21 to 0.08; I^2^ = 60.9%), at 1 week (MD −0.05 logMAR, 95% CI −0.11 to 0.02; I^2^ = 0%), or at 1 month (MD −0.03 logMAR, 95% CI −0.08 to 0.01; I^2^ = 0%). There was no significant difference between follow-up subgroups (*p* = 0.847; Figure 5).

##### Central Corneal Thickness (µm)

**Figure 6:**
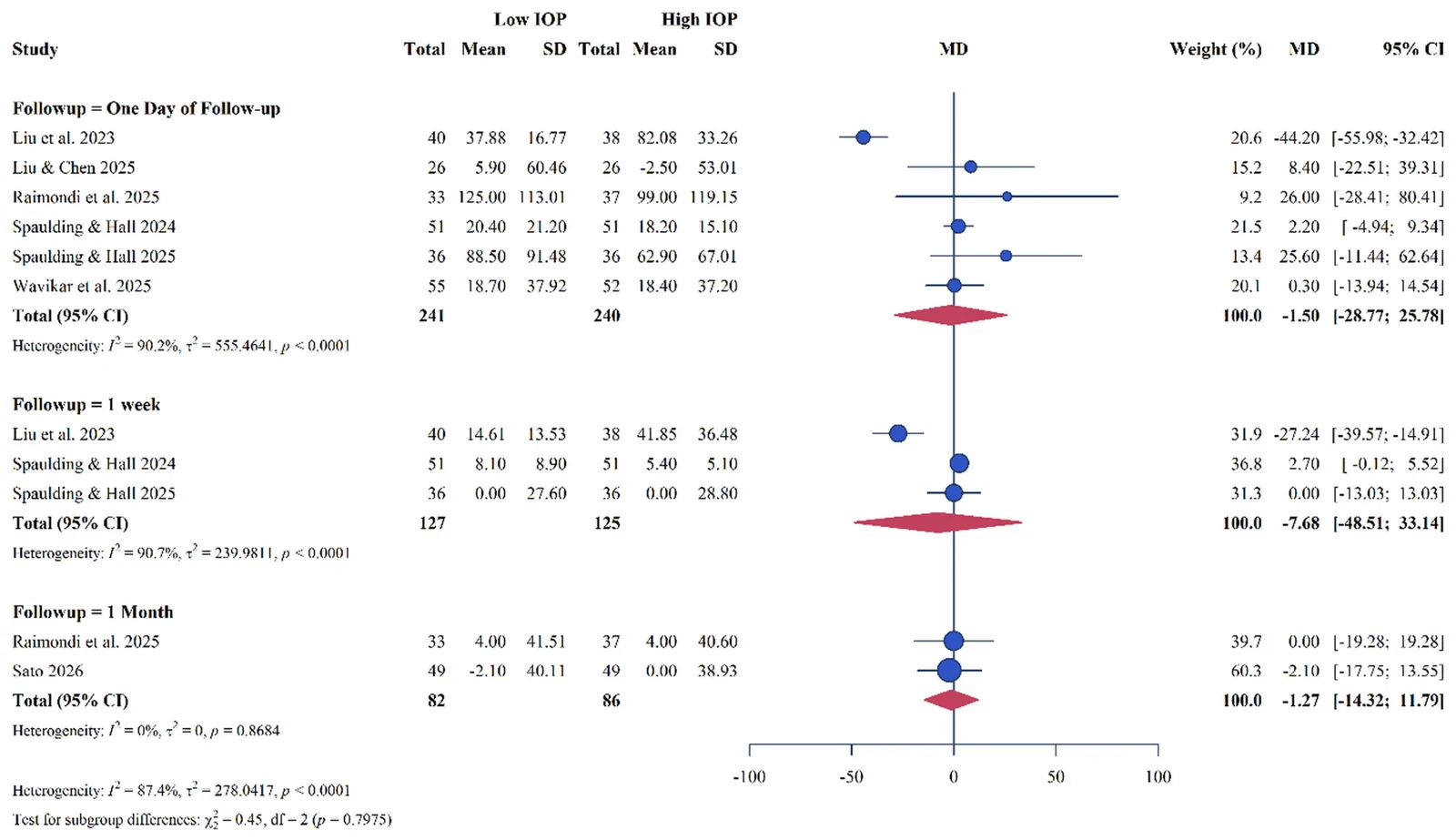
Forest plot of central corneal thickness (CCT), presented as mean differences (MDs) with corresponding 95% confidence intervals (CIs) [10,13,16,20,21,22,23].

Changes in central corneal thickness were also comparable between groups on postoperative day 1 (MD −1.50 µm, 95% CI −28.77 to 25.78; I^2^ = 90.2%), at 1 week (MD −7.68 µm, 95% CI −48.51 to 33.14; I^2^ = 90.7%), and at 1 month (MD −1.27 µm, 95% CI −14.32 to 11.79; I^2^ = 0%). The test for subgroup differences according to follow-up duration was not significant (*p* = 0.798; Figure 6).

#### 3.5.3. Postoperative Intraocular Pressure (mmHg)

Three studies reported postoperative changes in IOP. No significant difference was observed between low- and high-IOP strategies (MD −0.20 mmHg, 95% CI −1.27 to 0.86; *p* = 0.50; I^2^ = 0%; Figure S17). Leave-one-out sensitivity analysis did not materially alter the pooled estimate (Figure S18).

### 3.6. Safety Outcomes

There were no significant differences between the two studied groups regarding postoperative corneal edema (RR 1.00, 95% CI 0.14 to 6.99; *p* = 1.00; I^2^ = 0%). No posterior capsule rupture events were reported in either group across six studies (0/246 vs. 0/248). Postoperative IOP elevation was also comparable between groups (RR 1.23, 95% CI 0.56 to 2.73; *p* = 0.60; I^2^ = 0%), as shown in Figures S19–S21. Leave-one-out sensitivity analysis did not materially alter the pooled estimate in any outcome (Figures S22–S24).

## 4. Discussion

In this systematic review and meta-analysis of 12 RCTs involving 1051 eyes, low-IOP settings during phacoemulsification yielded broadly comparable intraoperative, postoperative, and safety outcomes to high-IOP settings. No significant between-group differences were observed in CDE, ECD across the evaluated postoperative time points, BCVA, CCT, postoperative IOP, operative efficiency, fluidics-related parameters, or safety outcomes. Although low-IOP phacoemulsification was associated with a statistically significant increase in total ultrasound time, the magnitude of this difference was small and unlikely to be clinically meaningful. Overall, these findings suggest that low-IOP strategies are feasible and provide clinical outcomes comparable to high-IOP strategies without an apparent increase in adverse outcomes across the available RCTs.

One of the principal findings of this analysis is that lowering intraoperative IOP did not compromise surgical performance. Traditionally, elevated irrigation pressures have been used to maintain anterior chamber stability, improve followability, and reduce the risk of post-occlusion surge during phacoemulsification [28]. However, modern advances in fluidics technology, particularly active-fluidics systems, have substantially improved the ability to maintain stable intraocular conditions across a wider range of pressure settings [29,30]. The absence of significant differences in CDE, operative duration, and fluid consumption suggests that contemporary phacoemulsification can be performed efficiently even at physiological IOP levels. Although total ultrasound time was statistically significantly longer in the low-IOP group, the absolute difference was only 1.53 s, which is unlikely to be clinically meaningful, particularly given the absence of a corresponding increase in CDE or measurable deterioration in postoperative outcomes [31]. Importantly, this finding was statistically fragile, as the pooled effect became non-significant in nearly all leave-one-out sensitivity analyses. Therefore, the observed increase in ultrasound time should be interpreted cautiously and may not represent a robust or clinically meaningful difference between the two strategies.

Low-IOP strategies have attracted increasing interest because of their potential to reduce pressure-related stress on ocular tissues [32]. High intraoperative IOP may transiently reduce ocular perfusion pressure and has therefore been hypothesized to contribute to pressure-related ocular stress, including endothelial injury, corneal edema, retinal perfusion disturbances, and patient discomfort [33,34]. Despite these theoretical advantages, our analysis did not demonstrate superior endothelial preservation, reduced corneal thickening, or faster visual recovery with physiological IOP. Several explanations may account for these findings. First, contemporary cataract surgery is generally associated with low levels of tissue trauma irrespective of the fluidics strategy employed [35]. Second, the duration of transient IOP elevation during routine phacoemulsification may be insufficient to produce measurable clinical consequences in most patients [36,37]. Importantly, many included trials primarily enrolled patients undergoing routine cataract surgery and did not specifically target eyes with glaucoma, endothelial dysfunction, impaired ocular perfusion, or other conditions that might increase susceptibility to transient IOP elevation. Consequently, the absence of a measurable benefit in the overall population should not necessarily be interpreted as evidence that lower intraoperative IOP has no value in higher-risk patients [10,13]. Finally, currently available trials may remain underpowered to identify small but clinically meaningful differences.

Current cataract surgery guidelines and expert recommendations primarily focus on maintaining surgical safety, efficiency, and chamber stability rather than targeting a specific intraoperative IOP level [38,39,40]. Consequently, surgeon preference and available fluidics technology continue to drive clinical practice. The findings of the present study suggest that low-IOP settings can be used without an apparent compromise in the measured surgical or postoperative outcomes evaluated in the available trials. However, the available evidence does not support routine use of low-IOP solely for the purpose of improving conventional postoperative outcomes. Rather, the choice of fluidics strategy may be individualized according to surgeon experience, equipment availability, and patient-specific considerations.

To our knowledge, this is the first systematic review and meta-analysis specifically focused on the clinical impact of different intraoperative IOP levels during phacoemulsification cataract surgery. Previous evidence syntheses have largely focused on comparisons between active-fluidics and gravity-fluidics systems, making it difficult to determine whether observed differences were attributable primarily to fluidics technology or to differences in intraoperative pressure settings [6,41]. By comparing low- versus high-IOP strategies, the present study provides insights into IOP management, although differences in fluidics technologies across some trials prevent definitive isolation of the independent effect of IOP. The findings suggest that although low-IOP is technically feasible and biologically attractive, its benefits may be more subtle than previously hypothesized and are not consistently reflected in conventional surgical or postoperative outcome measures.

The findings of this study have several practical implications for cataract surgeons. First, they suggest that lowering intraoperative IOP to physiological levels can be accomplished without compromising surgical efficiency or early postoperative outcomes. This may provide reassurance for surgeons utilizing active-fluidics systems or adopting lower-pressure surgical strategies. Second, the absence of clear superiority of low-IOP suggests that routine modification of surgical practice solely to achieve lower pressure targets may not be warranted based on currently available evidence. Finally, although average-risk patients appear to derive limited measurable benefit from low-IOP strategies, specific populations, including patients with glaucoma, impaired ocular perfusion, compromised endothelial reserve, or advanced vascular disease, may be more susceptible to the effects of transient IOP elevation. Whether these populations derive clinically meaningful benefit from lower IOP remains an important question for future randomized trials.

Several limitations should be considered when interpreting these findings. First, although all included studies were randomized trials, the overall sample size remained relatively modest for several individual outcomes. Consequently, the analyses may have been underpowered to detect small but clinically important differences. Moreover, the absence of statistically significant between-group differences should not be interpreted as evidence of equivalence, as the included trials were not necessarily designed or powered as formal equivalence or non-inferiority studies. Second, substantial heterogeneity was observed for selected outcomes, particularly endothelial cell density, central corneal thickness, intraoperative pain, and irrigation fluid usage. This heterogeneity may reflect differences in surgical platforms, fluidics systems, operative techniques, follow-up duration, and outcome assessment methods. For ECD specifically, additional sources of heterogeneity may include differences in baseline corneal endothelial status, cataract severity, and patient characteristics across studies. Notably, Liu et al. (2023) enrolled patients with reduced baseline ECD (500–1500 cells/mm^2^), whereas other trials generally included patients with relatively preserved endothelial function [16]. Such differences may contribute to variability in postoperative endothelial cell changes. Although subgroup analyses according to postoperative follow-up duration were conducted for ECD and CCT, substantial heterogeneity persisted at certain time points. Further subgroup analyses or meta-regression to explore other potential sources of heterogeneity were limited by the small number of studies contributing to individual outcomes. Therefore, these findings should be interpreted cautiously. Furthermore, percentage endothelial cell loss relative to baseline could not be consistently analyzed because the necessary outcome data were not uniformly available across studies. This may limit the clinical comparability of ECD changes between populations with different baseline endothelial cell counts. Third, interpretation of the findings is complicated by the fact that several trials compared interventions that differed not only in target IOP but also in the fluidics platform used. This limits our ability to isolate the independent effect of intraoperative IOP from the potential effects of the underlying fluidics technology. Moreover, differences in machine generation, active versus gravity-based fluidics systems, and associated surgical settings may have independently influenced surgical efficiency and postoperative outcomes. Consequently, the pooled estimates should be interpreted as reflecting the overall effects of low- versus high-IOP surgical strategies rather than the isolated physiological effect of intraoperative IOP. Furthermore, the definitions of low and high intraoperative IOP varied across studies, and the selected pressure ranges do not represent universally established clinical thresholds. Although the target pressure ranges were generally distinct, variability in actual intraoperative pressure and potential overlap between strategies may have reduced the contrast between groups and attenuated observable treatment effects. Although subgroup analysis was performed to explore the influence of fluidics platform differences, the limited number of studies in each subgroup restricts definitive conclusions regarding the independent effect of intraoperative IOP. Additionally, several included RCTs employed paired-eye designs. Because within-patient correlations were unavailable, these studies were analyzed without accounting for the paired nature of the data, potentially overestimating standard errors and yielding more conservative confidence intervals. Fourth, follow-up periods were generally short and varied across studies, limiting the assessment of potential long-term effects on corneal health, ocular perfusion, and visual function. Additionally, publication bias assessments should be interpreted cautiously, as Egger’s test has limited statistical power when the number of included studies is small. Therefore, non-significant test results do not necessarily exclude the possibility of publication bias or small-study effects. Finally, the use of aggregate study-level data precluded adjustment for individual patient characteristics and limited the ability to explore potentially important treatment-effect modifiers or identify patient subgroups that may derive greater benefit from low-IOP strategies.

Future research should focus on adequately powered, multicenter RCTs specifically designed to isolate the effect of intraoperative IOP while minimizing confounding from differences in fluidics platforms and surgical techniques. Where the primary objective is to establish whether low IOP is not clinically inferior to conventional higher-pressure strategies, appropriately designed non-inferiority or equivalence trials with prespecified clinically meaningful margins may be particularly informative. Studies enrolling patients with glaucoma, endothelial dysfunction, impaired ocular perfusion, ocular vascular disease, or other high-risk characteristics are also warranted, as these populations may be more susceptible to the potential adverse effects of transient intraoperative IOP elevation. Importantly, these high-risk populations may represent the groups in which the potential physiological advantages of low-IOP phacoemulsification are most clinically relevant, highlighting the need for dedicated trials evaluating patient-specific benefits rather than relying exclusively on findings from routine cataract surgery populations. Future investigations should additionally assess outcomes beyond conventional surgical metrics, including ocular perfusion parameters, endothelial cell loss, patient-reported discomfort, visual quality, and longer-term corneal and visual outcomes. Finally, evaluations of cost-effectiveness and resource utilization may help determine whether the potential technical and clinical benefits of low-IOP strategies justify their broader adoption in contemporary cataract surgery.

## 5. Conclusions

This systematic review and meta-analysis found no statistically significant differences between low- and high-IOP phacoemulsification in most intraoperative, postoperative, and safety outcomes. Despite a statistically significant but clinically negligible increase in ultrasound time of approximately 1.5 s, no significant differences were observed in CDE, ECD, BCVA, CCT, postoperative IOP, or safety outcomes. However, these findings should not be interpreted as definitive evidence of equivalence or non-inferiority between the two strategies. Low-IOP phacoemulsification appears to be a feasible alternative, although no clear clinical advantage was demonstrated. Further adequately powered RCTs are warranted to establish comparative effectiveness and identify patient subgroups that may derive greater benefit from low-IOP strategies.

## Figures and Tables

**Table 1:** Summary characteristics of the included randomized controlled trials.

Study ID	Study Design	Country	Sample Size	Population	Fluidics System		Surgical Technique	Follow-Up
					Low-IOP Arm	High-IOP Arm		
Georgiev et al., 2026 [27]	Single-center, self-control (paired-eye) RCT	Austria	54 eyes (27 patients): Low IOP = 27; High IOP = 27	Myopic patients with bilateral visually significant cataracts (AL > 25 mm)	AFS (Stellaris Elite), 20 mmHg	GFS, 100-cm bottle height	Standard phacoemulsification	Immediate; 1–2 weeks
Liu & Chen 2025 [13]	Single-center, parallel-group RCT	China	52 eyes (43 patients): Low IOP = 26; High IOP = 26	Patients with age-related cataract and high myopia (AL > 26 mm)	AFS + Active Sentry, 30 mmHg	GFS + OZil, 80 cmH_2_O	Standard phacoemulsification	1 month
Liu et al., 2023 [16]	Single-center, parallel-group RCT	China	78 eyes (53 patients): Low IOP = 40; High IOP = 38	Patients with senile cataract and reduced corneal ECD (500–1500 cells/mm^2^)	AFS + Active Sentry, 30 mmHg	GFS + OZil, 80 cmH_2_O	Standard phacoemulsification	1 month
Raimondi et al., 2025 [20]	Single-center, parallel-group RCT	UK	70 eyes (70 patients): Low IOP = 33; High IOP = 37	Cataract patients with diabetic retinopathy or primary open-angle glaucoma	AFS + Active Sentry, 30 mmHg	AFS + Active Sentry, 60 mmHg	Stop-and-chop	POD 1, 21, 40
Rauen et al., 2024 [25]	Single-center, self-control (paired-eye) RCT	USA	54 eyes (27 patients): Low IOP = 27; High IOP = 27	Patients with bilateral visually significant cataracts and otherwise healthy eyes	AFS + Active Sentry, ≤28 mmHg	AFS + Active Sentry, 65 mmHg	Horizontal phaco-chop	POD 1; 1 week; 1, 3 months
Sato 2026 [10]	Single-center, self-control (paired-eye) RCT	Japan	112 eyes (56 patients): Low IOP = 56; High IOP = 56	Non-diabetic patients undergoing routine bilateral cataract surgery	AFS + OZil (without Active Sentry), 28 mmHg	AFS + OZil (without Active Sentry), 55 mmHg	Eight-chop	POD 1; 1, 7, 19 weeks
Scarfone et al., 2024 [26]	Multicenter, parallel-group RCT	Argentina	80 eyes (80 patients): Low IOP = 40; High IOP = 40	Cataract patients aged 50–70 years with otherwise healthy eyes and no risk factors for anterior vitreous detachment	AFS + Active Sentry, 30 mmHg	AFS + Active Sentry, 80 mmHg	Stop-and-chop	POD 1, 7, 30, 90
Spaulding & Hall 2024 [21]	Single-center, self-control (contralateral-eye) RCT	USA	102 eyes (51 patients): Low IOP = 51; High IOP = 51	Adults undergoing uncomplicated bilateral cataract surgery with IOL implantation	Centurion + Active Sentry + Intrepid Hybrid Tip, 20 mmHg	Centurion + Active Sentry + Intrepid Hybrid Tip, 50–65 mmHg	Standard phacoemulsification	POD 1, 7
Spaulding & Hall 2025 [22]	Single-center, self-control (contralateral-eye) RCT	USA	72 eyes (36 patients): Low IOP = 36; High IOP = 36	Patients eligible for uncomplicated delayed sequential bilateral cataract surgery	Centurion + Active Sentry, 20 mmHg	Centurion + OZil, 50 mmHg	Groove-and-chop phacoemulsification	POD 1, 7
Vasavada et al., 2024 [19]	Single-center, parallel-group RCT	India	200 eyes (200 patients) overall; 160 eyes (160 patients) included: Low IOP = 80; High IOP = 80	Patients undergoing phacoemulsification for uncomplicated age-related cataract	Centurion AFS: traditional/Active Sentry, 30 mmHg	Centurion AFS: traditional/Active Sentry, 55 mmHg	Standardized phacoemulsification	POD 1; 1 week; 1, 3, 6 months
Wang et al., 2024 [24]	Single-center, parallel-group RCT	China	110 eyes (110 patients): Low IOP = 55; High IOP = 55	Patients with age-related cataract and otherwise healthy eyes	AFS + Active Sentry, 20 mmHg	AFS + Active Sentry, 60 mmHg	Phaco-chop	POD 1
Wavikar et al., 2025 [23]	Single-center, parallel-group RCT	India	107 eyes (107 patients): Low IOP = 55; High IOP = 52	Adults with immature NS2 cataract undergoing phacoemulsification	Centurion + Active Sentry, 20 mmHg	Centurion + Active Sentry, 50 mmHg	Direct chop	POD 1, 30, 90

Abbreviations: AFS, active fluidics system; AL, axial length; ECD, endothelial cell density; GFS, gravity fluidics system; IOL, intraocular lens; IOP, intraocular pressure; NS2, nuclear sclerosis grade 2; POD, postoperative day; RCT, randomized controlled trial; UK, United Kingdom; USA, United States of America.

**Table 2:** Baseline characteristics of the participants.

Study ID	Group/Arm	IOP Setting	Eyes	Patients	Age (Years) Mean (SD)	ACD (mm) Mean (SD)	CCT (µm) Mean (SD)	ECD (Cells/mm^2^) Mean (SD)	Male *n* (%)	Axial Length (mm) Mean (SD)	Laterality *n* (%)		Baseline BCVA (logMAR) Mean (SD)	Baseline IOP (mmHg) Mean (SD)
											Right	Left		
Georgiev et al., 2026 [27]	Low IOP	20 mmHg	27	27	66 (10)	3.45 (0.29)	NR	NR	NR	26.29 (0.86)	NR	NR	NR	NR
	High IOP	70 mm Hg	27	27		3.45 (0.25)	NR	NR	NR	26.26 (0.96)	NR	NR	NR	NR
Liu et al., 2023 [16]	Low IOP	30 mmHg	40	28	71.34 (7.52)	3.03 (0.34)	541.1 (35.2)	1213.6 (223.5)	15 (53.6)	NR	16 (40.0)	24 (60.0)	0.31 (0.25)	NR
	High IOP	59 mmHg	38	25	69.92 (6.89)	3.06 (0.52)	536.5 (31.2)	1256.1 (191.3)	12 (48.0)	NR	18 (47.4)	20 (52.6)	0.30 (0.25)	NR
Liu & Chen 2025 [13]	Low IOP	30 mmHg	26	20	66.15 (4.92)	3.51 (0.56)	554.1 (62.9)	2735.7 (409.5)	14 (53.8)	27.30 (1.59)	13 (50)	13 (50)	0.35 (0.19)	NR
	High IOP	59 mmHg	26	23	67.43 (5.77)	3.58 (0.61)	559.0 (57.8)	2895.2 (399.4)	13 (50)	27.49 (1.98)	15 (57.7)	11 (42.3)	0.33 (0.21)	NR
Raimondi et al., 2025 [20]	Low IOP	30 mmHg	33	33	70.3 (8.7)	3.0 (0.5)	541 (42)	NR	18 (55)	23.4 (1.5)	16 (48)	NR	0.58 (0.42)	16.5 (3.5)
	High IOP	60 mmHg	37	37	72.2 (9.4)	3.1 (0.4)	540 (36)	NR	16 (43)	23.8 (1.5)	25 (68)	NR	0.56 (0.40)	15.1 (3.2)
Rauen et al., 2024 [25]	Low IOP	≤28 mmHg	27	27	71	NR	550.0 (31.0)	2547.5 (367.7)	9 (33)	23.8 (1.2)	NR	NR	0.12 (0.10)	16.2 (4.0)
	High IOP	65 mmHg	27		71	NR	548.9 (29.7)	2566.9 (337.1)		23.8 (1.1)	NR	NR	0.14 (0.17)	15.8 (3.5)
Sato 2026 [10]	Low IOP	28 mmHg	56	56	73.4 (7.14)	3.21 (0.35)	530 (39.6)	2683 (228)	20 (35.7)	24.1 (1.64)	NR	NR	0.113 (0.131)	13.5 (1.60)
	High IOP	55 mmHg	56		73.4 (7.14)	3.21 (0.33)	530 (38.0)	2672 (235)		24.1 (1.59)	NR	NR	0.097 (0.214)	13.4 (1.84)
Scarfone et al., 2024 [26]	Low IOP	30 mmHg	40	40	66.82 (3.78)	3.14 (4.61)	525 (33.47)	NR	17 (42.5)	23.17 (0.72)	NR	NR	NR	NR
	High IOP	80 mmHg	40	40	66.09 (4.57)	3.20 (4.29)	528 (34.10)	NR	23 (57.5)	23.69 (0.75)	NR	NR	NR	NR
Spaulding & Hall 2024 [21]	Low IOP	20 mmHg	51	51	75.8 (6.7)	NR	547.1 (35.3)	NR	20 (39)	NR	NR	NR	NR	16.0 (2.8)
	High IOP	50–65 mmHg	51	51	75.8 (6.7)	NR	546.5 (36.2)	NR		NR	NR	NR	NR	16.5 (3.6)
Spaulding & Hall 2025 [22]	Low IOP	20 mmHg	36	36	71.3 (6.7)	NR	554.9 (27.6)	NR	17 (47)	NR	NR	NR	NR	NR
	High IOP	50 mmHg	36	36	71.3 (6.7)	NR	555.5 (28.8)	NR		NR	NR	NR	NR	NR
Vasavada et al., 2024 [19]	Low IOP	30 mmHg	40	40	NR	NR	NR	NR	NR	NR	NR	NR	NR	NR
	High IOP	55 mmHg	40	40	NR	NR	NR	NR	NR	NR	NR	NR	NR	NR
Wang et al., 2024 [24]	Low IOP	20 mmHg	55	55	68.73 (5.94)	NR	531.75 (26.36)	2676.62 (325.24)	25 (45.5)	NR	29 (52.7)	26 (47.3)	0.54 (0.32)	NR
	High IOP	60 mmHg	55	55	67.93 (6.75)	NR	533.27 (37.50)	2719.65 (51.66)	26 (47.3)	NR	27 (49.0)	28 (51.0)	0.55 (0.35)	NR
Wavikar et al., 2025 [23]	Low IOP	20 mmHg	55	55	65.3 (6.8)	3.18 (0.44)	511.9 (35.7)	2534.0 (200.7)	NR	NR	26 (47.3)	29 (52.7)	NR	NR
	High IOP	50 mmHg	52	52	64.8 (7.7)	3.09 (0.34)	521.1 (34.8)	2585.8 (234.7)	NR	NR	25 (48.1)	27 (51.9)	NR	NR

Abbreviations: ACD, anterior chamber depth; BCVA, best-corrected visual acuity; CCT, central corneal thickness; ECD, endothelial cell density; IOP, intraocular pressure; logMAR, logarithm of the minimum angle of resolution; mm, millimeter; mmHg, millimeters of mercury; NR, not reported; SD, standard deviation; µm, micrometer. Note: The unusually large standard deviations for anterior chamber depth reported by Scarfone et al., 2024 [26] and the unusually small standard deviation for endothelial cell density in the high-IOP group reported by Wang et al., 2024 [24] were reproduced as published in the original studies and may reflect reporting errors.

## Data Availability

No new data were created or analyzed in this study. Data sharing is not applicable to this article.

## Supplementary Materials

The following supporting information can be downloaded at: https://www.mdpi.com/article/10.3390/jcm15218557/s1, Table S1. The PRISMA 2020 checklist; Table S2. Search strategies and results across the electronic databases; Table S3. List of studies excluded after full-text assessment, with reasons for exclusion; Figure S1. Leave-one-out sensitivity analysis for cumulative dissipated energy (CDE); Figure S2. Funnel plot for cumulative dissipated energy (CDE); Figure S3. Subgroup analysis of cumulative dissipated energy (CDE) according to whether the comparison involved the same fluidics system (Active Fluidics System [AFS] vs. AFS) or different fluidics systems (AFS vs. Gravity Fluidics System [GFS]); Figure S4. Forest plot for intraoperative pain (VAS); Figure S5. Leave-one-out sensitivity analysis for intraoperative pain (VAS); Figure S6. Forest plot for aspiration time (seconds); Figure S7. Forest plot for total case time (seconds); Figure S8. Forest plot for total ultrasound time (seconds); Figure S9. Forest plot for irrigation fluid usage (mL); Figure S10. Forest plot for aspirated fluid volume (mL); Figure S11. Leave-one-out sensitivity analysis for aspiration time (seconds); Figure S12. Leave-one-out sensitivity analysis for total case time (seconds); Figure S13. Leave-one-out sensitivity analysis for total ultrasound time (seconds); Figure S14. Leave-one-out sensitivity analysis for irrigation fluid usage (mL); Figure S15. Leave-one-out sensitivity analysis for aspirated fluid volume (mL); Figure S16. Funnel plot for change in endothelial cell density (cells/mm^2^); Figure S17. Forest plot for change in postoperative intraocular pressure (mmHg); Figure S18. Leave-one-out sensitivity analysis for change in postoperative intraocular pressure (mmHg); Figure S19. Forest plot for postoperative corneal edema; Figure S20. Forest plot for posterior capsule rupture; Figure S21. Forest plot for postoperative IOP elevation; Figure S22. Leave-one-out sensitivity analysis for postoperative corneal edema; Figure S23. Leave-one-out sensitivity analysis for posterior capsule rupture; Figure S24. Leave-one-out sensitivity analysis for postoperative IOP elevation.
