# Supplementary material for: Low Versus High Intraoperative Intraocular Pressure During Phacoemulsification Cataract Surgery: A Systematic Review and Meta-Analysis of Randomized Controlled Trials
Source: J Clin Med. 2026 Nov 3;15(21):8557. doi: 10.3390/jcm15218557 (PMC13764359; doi:10.3390/jcm15218557)

SUPPLEMENTAL TABLE AND FIGURE LEGENDS

Table S1. The PRISMA 2020 checklist.

Table S2. Search strategies and results across the electronic databases.

Table S3. List of studies excluded after full-text assessment, with reasons for exclusion.

Figure S1. Leave-one-out sensitivity analysis for cumulative dissipated energy (CDE).

Figure S2. Funnel plot for cumulative dissipated energy (CDE).

Figure S3. Subgroup analysis of cumulative dissipated energy (CDE) according to whether the comparison involved the same fluidics system (Active Fluidics System [AFS] vs. AFS) or different fluidics systems (AFS vs. Gravity Fluidics System [GFS]).

Figure S4. Forest plot for intraoperative pain (VAS).

Figure S5. Leave-one-out sensitivity analysis for intraoperative pain (VAS).

Figure S6. Forest plot for aspiration time (seconds).

Figure S7. Forest plot for total case time (seconds).

Figure S8. Forest plot for total ultrasound time (seconds).

Figure S9. Forest plot for irrigation fluid usage (mL).

Figure S10. Forest plot for aspirated fluid volume (mL).

Figure S11. Leave-one-out sensitivity analysis for aspiration time (seconds).

Figure S12. Leave-one-out sensitivity analysis for total case time (seconds).

Figure S13. Leave-one-out sensitivity analysis for total ultrasound time (seconds).

Figure S14. Leave-one-out sensitivity analysis for irrigation fluid usage (mL).

Figure S15. Leave-one-out sensitivity analysis for aspirated fluid volume (mL).

Figure S16. Funnel plot for change in endothelial cell density (cells/mm^2).

Figure S17. Forest plot for change in postoperative intraocular pressure (mmHg).

Figure S18. Leave-one-out sensitivity analysis for change in postoperative intraocular pressure (mmHg).

Figure S19. Forest plot for postoperative corneal edema.

Figure S20. Forest plot for posterior capsule rupture.

Figure S21. Forest plot for postoperative IOP elevation.

Figure S22. Leave-one-out sensitivity analysis for postoperative corneal edema.

Figure S23. Leave-one-out sensitivity analysis for posterior capsule rupture.

Figure S24. Leave-one-out sensitivity analysis for postoperative IOP elevation.

Table S1. The PRISMA 2020 checklist.

Section and Topic	Item #	Checklist item	Location where item is reported
**TITLE**			
Title	1	Identify the report as a systematic review.	1
**ABSTRACT**			
Abstract	2	See the PRISMA 2020 for Abstracts checklist.	1
**INTRODUCTION**			
Rationale	3	Describe the rationale for the review in the context of existing knowledge.	2
Objectives	4	Provide an explicit statement of the objective(s) or question(s) the review addresses.	2
**METHODS**			
Eligibility criteria	5	Specify the inclusion and exclusion criteria for the review and how studies were grouped for the syntheses.	3
Information sources	6	Specify all databases, registers, websites, organisations, reference lists and other sources searched or consulted to identify studies. Specify the date when each source was last searched or consulted.	2-3
Search strategy	7	Present the full search strategies for all databases, registers and websites, including any filters and limits used.	2-3
Selection process	8	Specify the methods used to decide whether a study met the inclusion criteria of the review, including how many reviewers screened each record and each report retrieved, whether they worked independently, and if applicable, details of automation tools used in the process.	2-3
Data collection process	9	Specify the methods used to collect data from reports, including how many reviewers collected data from each report, whether they worked independently, any processes for obtaining or confirming data from study investigators, and if applicable, details of automation tools used in the process.	3
Data items	10a	List and define all outcomes for which data were sought. Specify whether all results that were compatible with each outcome domain in each study were sought (e.g. for all measures, time points, analyses), and if not, the methods used to decide which results to collect.	3
	10b	List and define all other variables for which data were sought (e.g. participant and intervention characteristics, funding sources). Describe any assumptions made about any missing or unclear information.	3
Study risk of bias assessment	11	Specify the methods used to assess risk of bias in the included studies, including details of the tool(s) used, how many reviewers assessed each study and whether they worked independently, and if applicable, details of automation tools used in the process.	3
Effect measures	12	Specify for each outcome the effect measure(s) (e.g. risk ratio, mean difference) used in the synthesis or presentation of results.	4
Synthesis methods	13a	Describe the processes used to decide which studies were eligible for each synthesis (e.g. tabulating the study intervention characteristics and comparing against the planned groups for each synthesis (item #5)).	4
	13b	Describe any methods required to prepare the data for presentation or synthesis, such as handling of missing summary statistics, or data conversions.	4
	13c	Describe any methods used to tabulate or visually display results of individual studies and syntheses.	4
	13d	Describe any methods used to synthesize results and provide a rationale for the choice(s). If meta-analysis was performed, describe the model(s), method(s) to identify the presence and extent of statistical heterogeneity, and software package(s) used.	4
	13e	Describe any methods used to explore possible causes of heterogeneity among study results (e.g. subgroup analysis, meta-regression).	4
	13f	Describe any sensitivity analyses conducted to assess robustness of the synthesized results.	4
Reporting bias assessment	14	Describe any methods used to assess risk of bias due to missing results in a synthesis (arising from reporting biases).	4
Certainty assessment	15	Describe any methods used to assess certainty (or confidence) in the body of evidence for an outcome.	N/A

Section and Topic	Item #	Checklist item	Location where item is reported
**RESULTS**			
Study selection	16a	Describe the results of the search and selection process, from the number of records identified in the search to the number of studies included in the review, ideally using a flow diagram.	4
	16b	Cite studies that might appear to meet the inclusion criteria, but which were excluded, and explain why they were excluded.	4
Study characteristics	17	Cite each included study and present its characteristics.	5-6, Table 1 and 2
Risk of bias in studies	18	Present assessments of risk of bias for each included study.	8, Figure 2
Results of individual studies	19	For all outcomes, present, for each study: (a) summary statistics for each group (where appropriate) and (b) an effect estimate and its precision (e.g. confidence/credible interval), ideally using structured tables or plots.	11, 12, Figure 2
Results of syntheses	20a	For each synthesis, briefly summarise the characteristics and risk of bias among contributing studies.	9-13
	20b	Present results of all statistical syntheses conducted. If meta-analysis was done, present for each the summary estimate and its precision (e.g. confidence/credible interval) and measures of statistical heterogeneity. If comparing groups, describe the direction of the effect.	9-13
	20c	Present results of all investigations of possible causes of heterogeneity among study results.	9-13
	20d	Present results of all sensitivity analyses conducted to assess the robustness of the synthesized results.	9-13
Reporting biases	21	Present assessments of risk of bias due to missing results (arising from reporting biases) for each synthesis assessed.	8, Figure 2
Certainty of evidence	22	Present assessments of certainty (or confidence) in the body of evidence for each outcome assessed.	NA
**DISCUSSION**			
Discussion	23a	Provide a general interpretation of the results in the context of other evidence.	13-14
	23b	Discuss any limitations of the evidence included in the review.	14-15
	23c	Discuss any limitations of the review processes used.	14-15
	23d	Discuss implications of the results for practice, policy, and future research.	15-16
**OTHER INFORMATION**			
Registration and protocol	24a	Provide registration information for the review, including register name and registration number, or state that the review was not registered.	2
	24b	Indicate where the review protocol can be accessed, or state that a protocol was not prepared.	2
	24c	Describe and explain any amendments to information provided at registration or in the protocol.	2
Support	25	Describe sources of financial or non-financial support for the review, and the role of the funders or sponsors in the review.	17
Competing interests	26	Declare any competing interests of review authors.	17
Availability of data, code and other materials	27	Report which of the following are publicly available and where they can be found: template data collection forms; data extracted from included studies; data used for all analyses; analytic code; any other materials used in the review.	17

Table S2. Search strategies and results across the electronic databases.

Database	Search query	Filter	Search results
**PubMed**	(("Phacoemulsification"[Mesh] OR "Cataract Extraction"[Mesh] OR phacoemulsification OR phaco OR "cataract surgery" OR "cataract extraction") AND ("Intraocular Pressure"[Mesh] OR "intraocular pressure" OR IOP OR "target IOP" OR "low IOP" OR "high IOP" OR "physiologic IOP" OR "physiological IOP" OR fluidics OR "active fluidics" OR "infusion pressure" OR "irrigation pressure" OR "bottle height" OR "anterior chamber pressure"))	Randomized Controlled Trial	N = 776
**Scopus**	TITLE-ABS-KEY(phacoemulsification OR phaco OR "cataract surgery" OR "cataract extraction") AND TITLE-ABS-KEY("intraocular pressure" OR IOP OR "target IOP" OR "low IOP" OR "high IOP" OR "physiologic IOP" OR "physiological IOP" OR fluidics OR "active fluidics" OR "infusion pressure" OR "irrigation pressure" OR "bottle height" OR "anterior chamber pressure") AND TITLE-ABS-KEY(randomized OR randomised OR randomly OR trial OR "controlled trial" OR "clinical trial" OR "contralateral eye" OR "paired-eye" OR "paired eye")	Article title, Abstract, Keywords	N = 2,832
**Web of Science (WoS)**	TS=(phacoemulsification OR phaco OR "cataract surgery" OR "cataract extraction") AND TS=("intraocular pressure" OR IOP OR "target IOP" OR "low IOP" OR "high IOP" OR "physiologic IOP" OR "physiological IOP" OR fluidics OR "active fluidics" OR "infusion pressure" OR "irrigation pressure" OR "bottle height" OR "anterior chamber pressure") AND TS=(randomized OR randomised OR randomly OR trial OR "controlled trial" OR "clinical trial" OR "contralateral eye" OR "paired-eye" OR "paired eye")	Topic	N = 1,542
**Cochrane Cochrane Central Register of Controlled Trials (CENTRAL)**	(phacoemulsification OR phaco OR "cataract surgery" OR "cataract extraction") AND ("intraocular pressure" OR IOP OR "target IOP" OR "low IOP" OR "high IOP" OR "physiologic IOP" OR "physiological IOP" OR fluidics OR "active fluidics" OR "infusion pressure" OR "irrigation pressure" OR "bottle height" OR "anterior chamber pressure")	Title, Abstract, Keyword	N = 2,076
**Total**			N = 7,266
**After Duplicate Removing (By EndNote 21)**			N = 4,951

Table S3. List of studies excluded after full-text assessment, with reasons for exclusion.

Study	Design	Settings	Primary eligibility rationale
**Zhao et al. 2021**	Randomized paired-eye AFS vs GFS	50 mmHg vs 65 mmHg	Low-pressure arm outside predefined physiological range
**Scampoli et al. 2026**	Randomized AFS vs GFS	40 mmHg vs 65 mmHg	Low-pressure arm outside predefined physiological range
**Luo et al. 2022**	Randomized AFS vs GFS	50 mmHg vs 65 mmHg	Low-pressure arm outside predefined physiological range
**Malik et al. 2017**	Randomized 3-group infusion/tip comparison	55 mmHg vs 81 mmHg	No physiological 20–30-mmHg intervention arm
**Solomon et al. 2016**	Randomized paired-eye Active Fluidics configuration vs Infiniti gravity configuration	Randomized system configuration, not an eligible low target IOP demonstrated	No defined eligible physiological vs elevated IOP contrast
**Gonzalez-Salinas et al. 2018**	Random assignment to Centurion Active Fluidics vs Infiniti gravity platform	Eligible 20–30-mmHg low target not documented	Platform comparison without a demonstrated physiological low-IOP strategy
**Chang et al. 2017**	Randomized low-fluidics vs standard-fluidics package	Bottle heights vary by phase: low 40/50/70 cm vs standard 95/100/100 cm, with simultaneous flow/vacuum changes	No defined physiological low-IOP strategy; intervention was a multicomponent low-fluidics regimen

Figure S1. Leave-one-out sensitivity analysis for cumulative dissipated energy (CDE).

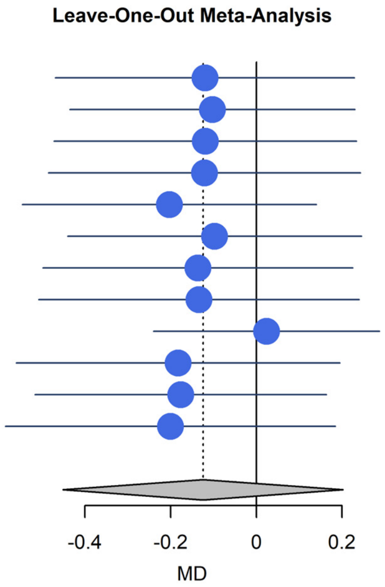

Study	MD	95%-CI	P-value	Tau2	Tau	I2
Omitting Liu & Chen 2025	-0.12	[-0.47; 0.23]	0.4608	0.0625	0.2500	19.1%
Omitting Liu et al. 2023	-0.10	[-0.44; 0.23]	0.5078	0.0539	0.2322	13.0%
Omitting Raimondi et al. 2025	-0.12	[-0.47; 0.23]	0.4693	0.0670	0.2588	18.9%
Omitting Rauen et al. 2024	-0.12	[-0.49; 0.24]	0.4754	0.0800	0.2828	18.7%
Omitting Sato 2026	-0.20	[-0.55; 0.14]	0.2172	0.0621	0.2491	6.2%
Omitting Scarfone et al. 2024	-0.10	[-0.44; 0.25]	0.5405	0.0570	0.2388	14.5%
Omitting Spaulding & Hall 2024	-0.14	[-0.50; 0.22]	0.4191	0.0797	0.2823	19.9%
Omitting Spaulding & Hall 2025	-0.13	[-0.51; 0.24]	0.4435	0.0945	0.3074	19.3%
Omitting Vasavada et al. 2024	0.02	[-0.24; 0.29]	0.8600	0	0	0%
Omitting Vasavada et al. 2024	-0.18	[-0.56; 0.19]	0.3063	0.1046	0.3235	18.0%
Omitting Wang et al. 2024	-0.18	[-0.52; 0.16]	0.2742	0.0740	0.2719	12.2%
Omitting Wavikar et al. 2025	-0.20	[-0.59; 0.18]	0.2725	0.1040	0.3226	14.6%
**Random effects model (HK-IQ)**	**-0.12**	**[-0.45; 0.20]**	**0.4196**	**0.0591**	**0.2430**	**11.9%**

Figure S2. Funnel plot for cumulative dissipated energy (CDE).

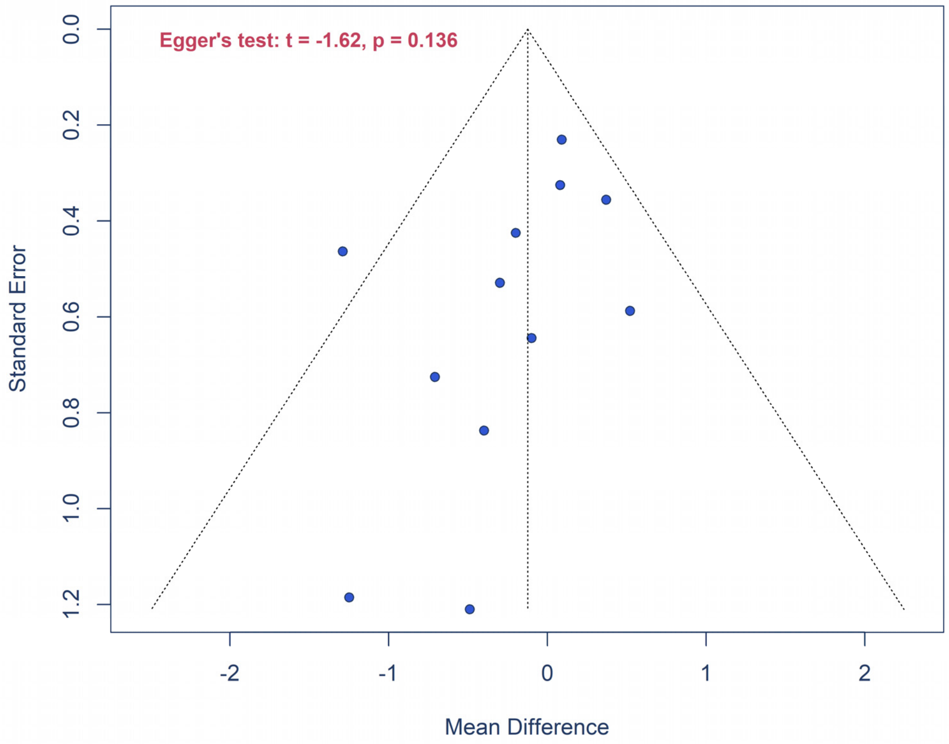

Figure S3. Subgroup analysis of cumulative dissipated energy (CDE) according to whether the comparison involved the same fluidics system (Active Fluidics System [AFS] vs. AFS) or different fluidics systems (AFS vs. Gravity Fluidics System [GFS]).

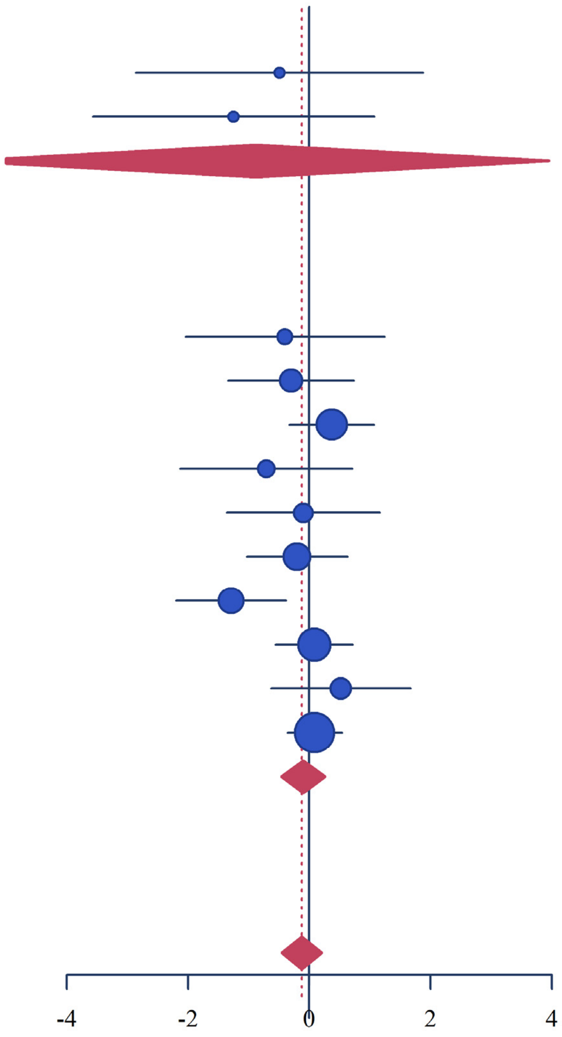

	Low IOP			High IOP					
**Study**	**Total**	**Mean**	**SD**	**Total**	**Mean**	**SD**	**Weight (%)**	**MD**	**95% CI**
**Comparison = AFS vs GFS**									
Liu & Chen 2025	26	5.47	3.72	26	5.96	4.92	1.6	-0.49	[-2.86; 1.88]
Liu et al. 2023	40	5.52	4.91	38	6.77	5.52	1.7	-1.25	[-3.57; 1.07]
**Total (95% CI)**	**66**			**64**			**3.3**	**-0.88**	**[-5.71; 3.95]**
Heterogeneity: $I^2 = 0\%$, $\tau^2 = 0$, $p = 0.6536$									
Test for effect in subgroup: $t_1 = -2.31$ ($p = 0.2600$)									
**Comparison = AFS vs AFS**									
Raimondi et al. 2025	33	6.10	3.40	37	6.50	3.60	3.3	-0.40	[-2.04; 1.24]
Rauen et al. 2024	27	2.85	2.23	27	3.15	1.61	7.4	-0.30	[-1.34; 0.74]
Sato 2026	56	5.93	1.87	56	5.56	1.90	13.4	0.37	[-0.33; 1.07]
Scarfone et al. 2024	40	7.31	3.19	40	8.02	3.30	4.3	-0.71	[-2.13; 0.71]
Spaulding & Hall 2024	51	8.60	3.40	51	8.70	3.10	5.3	-0.10	[-1.36; 1.16]
Spaulding & Hall 2025	36	5.00	1.90	36	5.20	1.70	10.4	-0.20	[-1.03; 0.63]
Vasavada et al. 2024	40	3.47	1.96	40	4.76	2.18	9.1	-1.29	[-2.20; -0.38]
Vasavada et al. 2024	40	3.00	1.40	40	2.92	1.51	15.1	0.08	[-0.56; 0.72]
Wang et al. 2024	55	5.22	3.31	55	4.70	2.83	6.2	0.52	[-0.63; 1.67]
Wavikar et al. 2025	55	6.11	1.28	52	6.02	1.10	22.3	0.09	[-0.36; 0.54]
**Total (95% CI)**	**433**			**434**			**96.7**	**-0.10**	**[-0.46; 0.26]**
Heterogeneity: $I^2 = 20.8\%$, $\tau^2 = 0.0574$, $p = 0.2513$									
Test for effect in subgroup: $t_9 = -0.62$ ($p = 0.5520$)									
**Total (95% CI)**	**499**			**498**			**100.0**	**-0.12**	**[-0.45; 0.20]**

Test for subgroup differences:

Chi-squared = 3.59, df = 1 (p = 0.0580)

Figure S4. Forest plot for intraoperative pain (VAS).

Study	Low IOP Events	Low IOP Total	High IOP Events	High IOP Total	Weight (%)	RR	95% CI
Liu & Chen 2025	5	26	18	26	29.7	0.28	[0.12; 0.64]
Liu et al. 2023	7	40	27	38	31.1	0.25	[0.12; 0.50]
Scarfone et al. 2024	0	40	32	40	11.2	0.02	[0.00; 0.24]
Spaulding & Hall 2024	7	51	7	51	28.0	1.00	[0.38; 2.65]
**Total (95% CI)**		**157**		**155**	**100.0**	**0.28**	**[0.03; 2.44]**

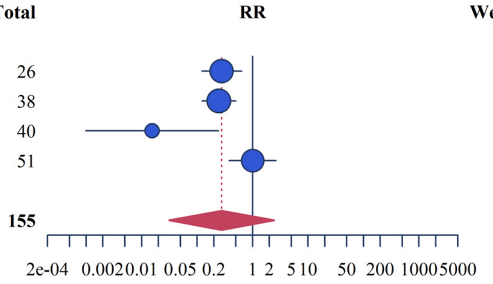

Heterogeneity: $I^2 = 71.6\%$, $\tau^2 = 0.9170$, $p = 0.0144$

Test for overall effect: $t_3 = -1.88$ ($p = 0.1569$)

Figure S5. Leave-one-out sensitivity analysis for intraoperative pain (VAS).

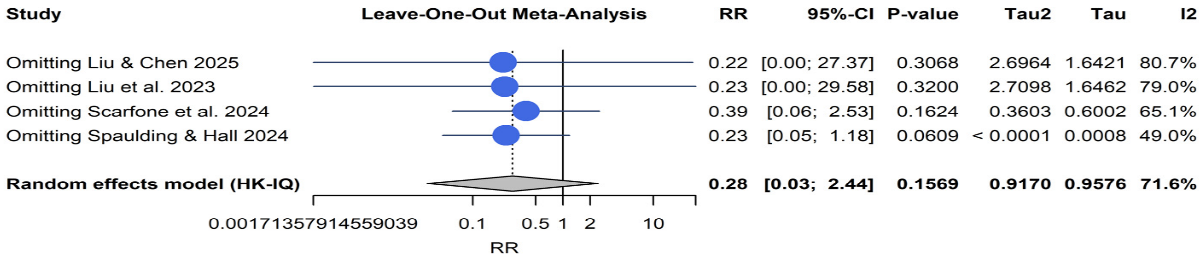

Figure S6. Forest plot for aspiration time (seconds).

Study	Low IOP Total	Low IOP Mean	Low IOP SD	High IOP Total	High IOP Mean	High IOP SD	Weight (%)	MD	95% CI
Rauen et al. 2024	27	105.00	21.82	27	112.80	21.82	14.4	-7.80	[-19.44; 3.84]
Sato 2026	56	75.00	18.30	56	69.00	17.90	33.4	6.00	[-0.70; 12.70]
Scarfone et al. 2024	40	211.47	40.66	40	191.18	41.40	6.6	20.29	[2.31; 38.27]
Spaulding & Hall 2024	51	106.70	25.30	51	101.70	27.20	18.0	5.00	[-5.20; 15.20]
Spaulding & Hall 2025	36	90.10	16.40	36	85.10	17.00	27.6	5.00	[-2.72; 12.72]
**Total (95% CI)**	**210**			**210**			**100.0**	**4.50**	**[-4.20; 13.20]**

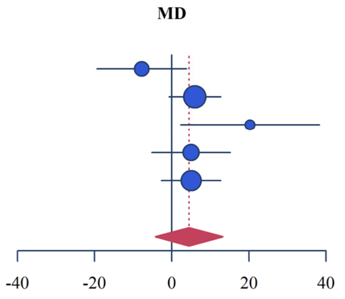

Heterogeneity: $I^2 = 46.4\%$, $\tau^2 = 6.2743$, $p = 0.1131$

Test for overall effect: $t_4 = 1.44$ ($p = 0.2243$)

Figure S7. Forest plot for total case time (seconds).

	Low IOP			High IOP					
**Study**	**Total**	**Mean**	**SD**	**Total**	**Mean**	**SD**	**Weight (%)**	**MD**	**95% CI**
Liu & Chen 2025	26	390.40	91.40	26	405.40	104.50	10.9	-15.00	[-68.36; 38.36]
Liu et al. 2023	40	323.50	151.70	38	372.30	169.80	7.1	-48.80	[-120.39; 22.79]
Raimondi et al. 2025	33	408.00	55.80	37	457.80	138.60	12.4	-49.80	[-98.35; -1.25]
Rauen et al. 2024	27	312.00	52.98	27	314.40	40.53	23.3	-2.40	[-27.56; 22.76]
Sato 2026	56	304.80	66.00	56	289.20	67.80	23.5	15.60	[-9.18; 40.38]
Spaulding & Hall 2024	51	267.90	71.40	51	246.90	62.90	22.7	21.00	[-5.12; 47.12]
**Total (95% CI)**	**233**			**235**			**100.0**	**-3.38**	**[-32.65; 25.89]**

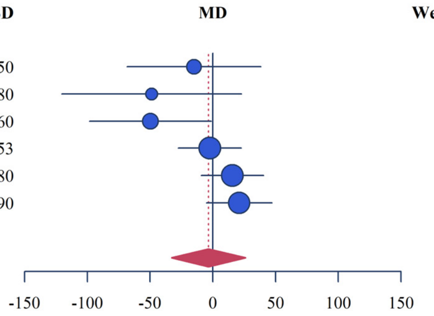

Heterogeneity: I^2 = 49.9%, τ^2 = 345.1026, p = 0.0756

Test for overall effect: t_5 = -0.30 (p = 0.7784)

Figure S8. Forest plot for total ultrasound time (seconds).

Study	Low IOP Total	Low IOP Mean	Low IOP SD	High IOP Total	High IOP Mean	High IOP SD	Weight (%)	MD	95% CI
Raimondi et al. 2025	33	35.33	19.38	37	34.33	17.06	2.1	1.00	[-7.60; 9.60]
Sato 2026	56	16.20	5.22	56	13.90	4.40	42.1	2.30	[0.51; 4.09]
Scarfone et al. 2024	40	75.70	25.89	40	68.33	21.07	1.4	7.37	[-2.97; 17.71]
Spaulding & Hall 2024	51	41.80	10.20	51	41.60	10.00	9.8	0.20	[-3.72; 4.12]
Spaulding & Hall 2025	36	29.50	7.30	36	31.00	7.40	12.9	-1.50	[-4.90; 1.90]
Wang et al. 2024	55	30.60	15.06	55	27.39	13.75	5.2	3.21	[-2.18; 8.60]
Wavikar et al. 2025	55	40.51	6.51	52	38.86	5.71	26.5	1.65	[-0.67; 3.97]
**Total (95% CI)**	**326**			**327**			**100.0**	**1.53**	**[0.03; 3.03]**

Heterogeneity: $I^2 = 0.0\%$, $\tau^2 = 0.1226$, $p = 0.4420$

Test for overall effect: $t_6 = 2.49$ ($p = 0.0470$)

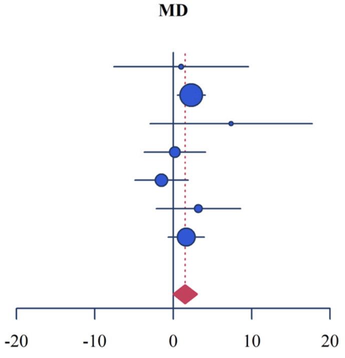

Figure S9. Forest plot for irrigation fluid usage (mL).

Study	Low IOP Total	Low IOP Mean	Low IOP SD	High IOP Total	High IOP Mean	High IOP SD	Weight (%)	MD	95% CI
Liu et al. 2023	40	38.00	19.80	38	41.20	28.50	8.8	-3.20	[-14.14; 7.74]
Rauen et al. 2024	27	40.00	11.95	27	55.60	9.87	13.0	-15.60	[-21.45; -9.75]
Sato 2026	56	26.60	7.71	56	25.20	7.35	15.2	1.40	[-1.39; 4.19]
Spaulding & Hall 2024	51	40.20	7.80	51	41.60	9.00	14.9	-1.40	[-4.67; 1.87]
Spaulding & Hall 2025	36	32.80	4.60	36	33.80	6.00	15.3	-1.00	[-3.47; 1.47]
Vasavada et al. 2024 (Traditional)	40	54.30	15.79	40	72.60	38.58	7.5	-18.30	[-31.22; -5.38]
Vasavada et al. 2024 (Active Sentry)	40	54.90	15.52	40	60.05	19.13	11.4	-5.15	[-12.78; 2.48]
Wang et al. 2024	55	45.07	12.68	55	42.38	11.93	14.0	2.69	[-1.91; 7.29]
**Total (95% CI)**	**345**			**343**			**100.0**	**-4.04**	**[-10.10; 2.02]**

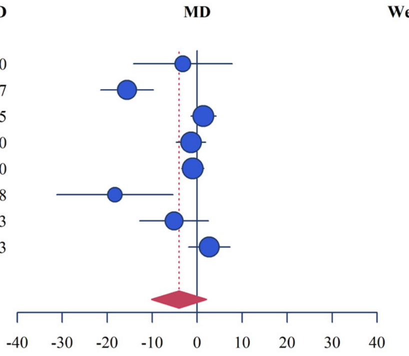

Heterogeneity: $I^2 = 81.2\%$, $\tau^2 = 38.5373$, $p < 0.0001$

Test for overall effect: $t_7 = -1.58$ ($p = 0.1592$)

Figure S10. Forest plot for aspirated fluid volume (mL).

	Low IOP			High IOP						
**Study**	**Total**	**Mean**	**SD**	**Total**	**Mean**	**SD**	**MD**	**Weight (%)**	**MD**	**95% CI**
Raimondi et al. 2025	33	46.33	10.08	37	48.33	13.12		29.0	-2.00	[-7.45; 3.45]
Rauen et al. 2024	27	33.60	7.27	27	40.10	7.27		36.8	-6.50	[-10.38; -2.62]
Scarfone et al. 2024	40	51.92	9.83	40	51.04	10.21		34.2	0.88	[-3.51; 5.27]
**Total (95% CI)**	**100**			**104**				**100.0**	**-2.67**	**[-12.21; 6.87]**

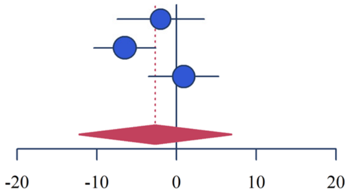

Heterogeneity: $I^2 = 68.0\%$, $\tau^2 = 10.3945$, $p = 0.0438$

Test for overall effect: $t_2 = -1.21$ ($p = 0.3514$)

Figure S11. Leave-one-out sensitivity analysis for aspiration time (seconds).

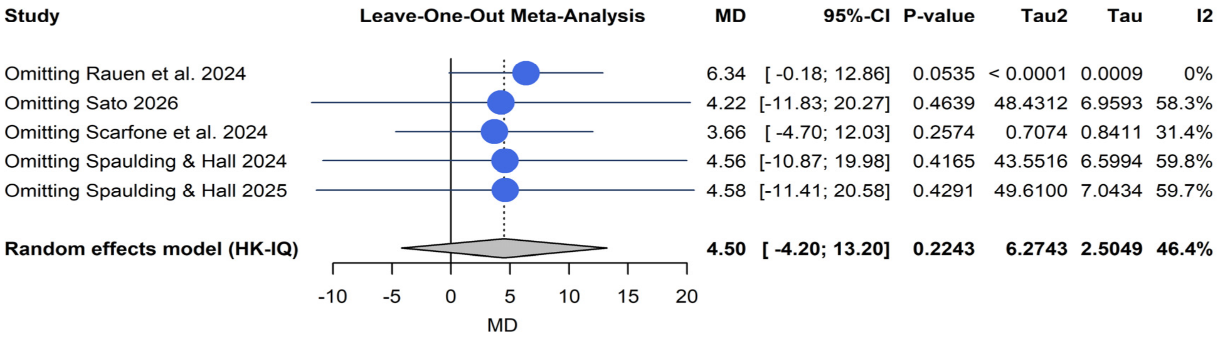

Figure S12. Leave-one-out sensitivity analysis for total case time (seconds).

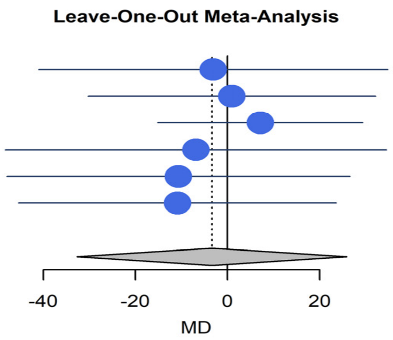

Study	MD	95%-CI	P-value	Tau2	Tau	I2
Omitting Liu & Chen 2025	-3.11	[-41.02; 34.80]	0.8311	462.9312	21.5158	58.0%
Omitting Liu et al. 2023	0.92	[-30.29; 32.13]	0.9387	249.8394	15.8063	49.4%
Omitting Raimondi et al. 2025	7.12	[-15.13; 29.37]	0.4244	4.8733	2.2076	21.4%
Omitting Rauen et al. 2024	-6.90	[-48.28; 34.48]	0.6675	668.7262	25.8597	59.0%
Omitting Sato 2026	-10.68	[-47.92; 26.57]	0.4707	495.1208	22.2513	53.3%
Omitting Spaulding & Hall 2024	-10.89	[-45.37; 23.59]	0.4300	375.5586	19.3793	46.7%
**Random effects model (HK-IQ)**	**-3.38**	**[-32.65; 25.89]**	**0.7784**	**345.1026**	**18.5769**	**49.9%**

Figure S13. Leave-one-out sensitivity analysis for total ultrasound time (seconds).

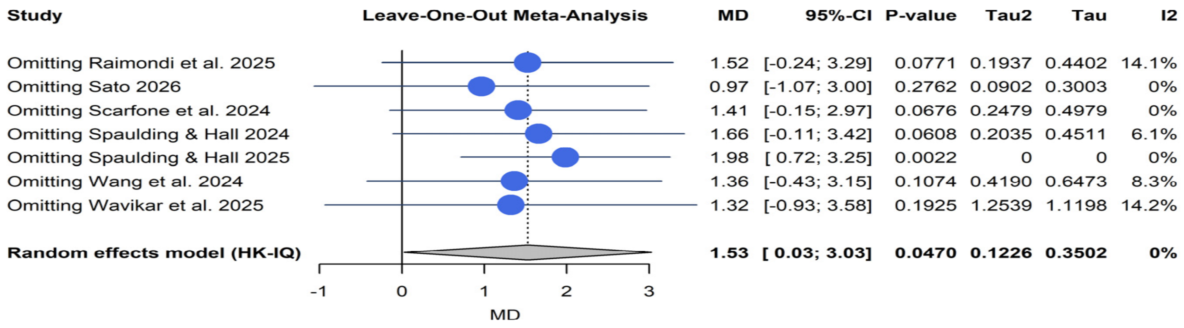

Figure S14. Leave-one-out sensitivity analysis for irrigation fluid usage (mL).

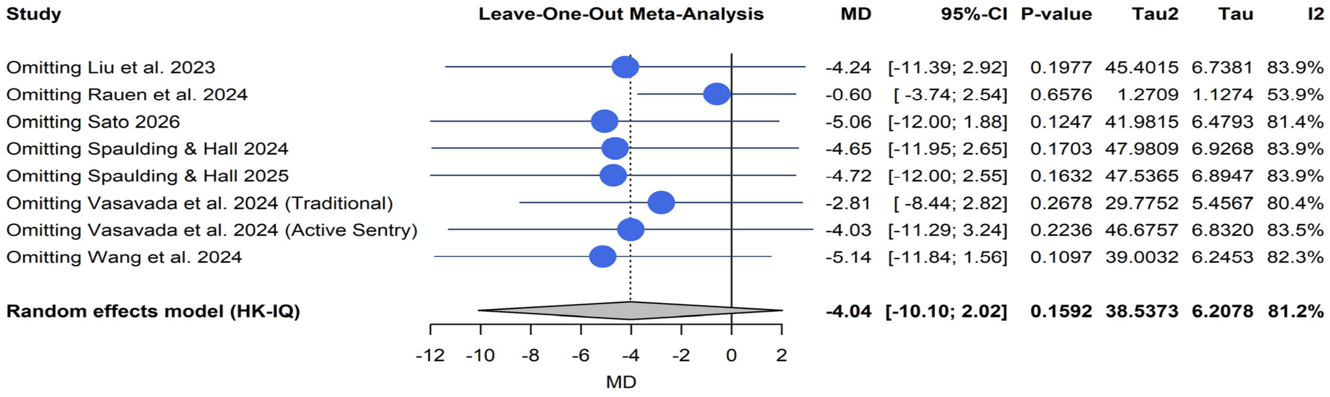

Figure S15. Leave-one-out sensitivity analysis for aspirated fluid volume (mL).

Study	MD	95%-CI	P-value	Tau2	Tau	I2
Omitting Raimondi et al. 2025	-2.88	[-49.76; 43.99]	0.5775	22.7638	4.7711	83.6%
Omitting Rauen et al. 2024	-0.25	[-18.13; 17.63]	0.8863	0	0	0%
Omitting Scarfone et al. 2024	-4.67	[-32.75; 23.40]	0.2811	4.3019	2.0741	42.5%
**Random effects model (HK-IQ)**	**-2.67**	**[-12.21; 6.87]**	**0.3514**	**10.3945**	**3.2241**	**68.0%**

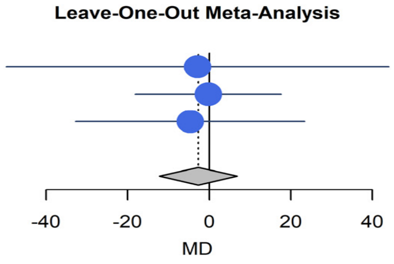

Figure S16. Funnel plot for change in endothelial cell density (cells/mm²).

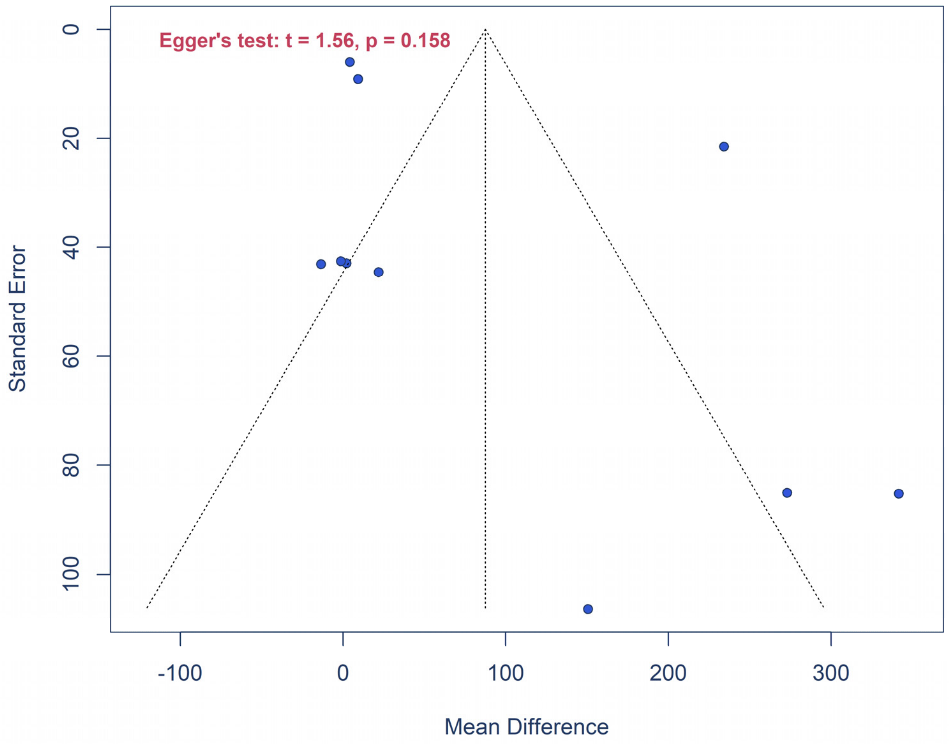

Figure S17. Forest plot for change in postoperative intraocular pressure (mmHg).

Study	Low IOP			High IOP			MD	Weight (%)	MD	95% CI
	Total	Mean	SD	Total	Mean	SD				
Raimondi et al. 2025	33	-1.70	3.50	37	-0.80	3.36		15.0	-0.90	[-2.51; 0.71]
Sato 2026	43	-1.15	1.75	43	-0.95	1.91		65.0	-0.20	[-0.97; 0.57]
Spaulding & Hall 2024	51	-0.80	3.41	51	-1.10	3.76		20.1	0.30	[-1.09; 1.69]
**Total (95% CI)**	**127**			**131**				**100.0**	**-0.20**	**[-1.27; 0.86]**

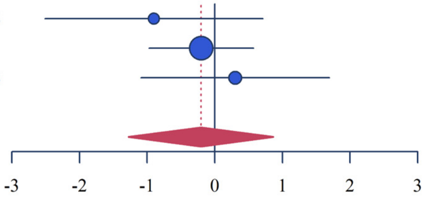

Heterogeneity: $I^2 = 0.0\%$, $\tau^2 = 0$, $p = 0.5436$

Test for overall effect: $t_2 = -0.82$ ($p = 0.4969$)

Figure S18. Leave-one-out sensitivity analysis for change in postoperative intraocular pressure (mmHg).

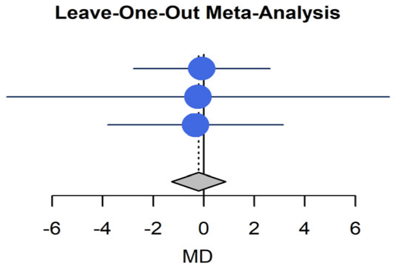

Study	MD	95%-CI	P-value	Tau2	Tau	I2
Omitting Raimondi et al. 2025	-0.08	[-2.78; 2.61]	0.7651	0	0	0%
Omitting Sato 2026	-0.23	[-7.80; 7.34]	0.7666	0.1292	0.3594	17.9%
Omitting Spaulding & Hall 2024	-0.33	[-3.80; 3.14]	0.4391	0	0	0%
**Random effects model (HK-IQ)**	**-0.20**	**[-1.27; 0.86]**	**0.4969**	**0**	**0**	**0%**

Figure S19. Forest plot for postoperative corneal edema.

Study	Low IOP Events	Low IOP Total	High IOP Events	High IOP Total	Weight (%)	RR	95% CI
Liu et al. 2023	1	40	1	38	50.4	0.95	[0.06; 14.65]
Raimondi et al. 2025	0	33	0	37	24.9	1.12	[0.02; 54.87]
Sato 2026	0	56	0	56	24.8	1.00	[0.02; 49.53]
**Total (95% CI)**		**129**		**131**	**100.0**	**1.00**	**[0.14; 6.99]**

Heterogeneity: $I^2 = 0.0\%$, $\tau^2 = 0$, $p = 0.9977$

Test for overall effect: $t_{Inf} = 0.00$ ($p = 0.9982$)

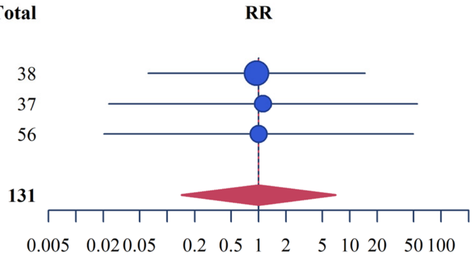

Figure S20. Forest plot for posterior capsule rupture.

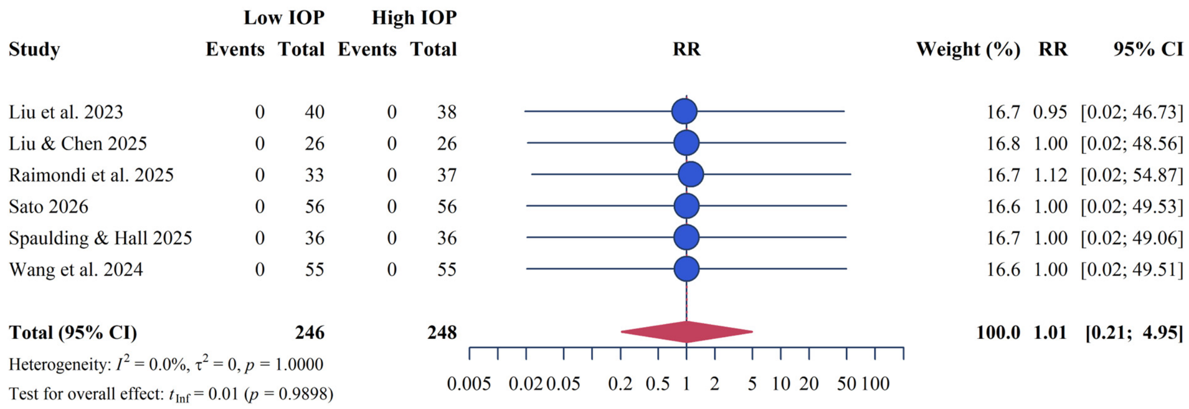

Figure S21. Forest plot for postoperative IOP elevation.

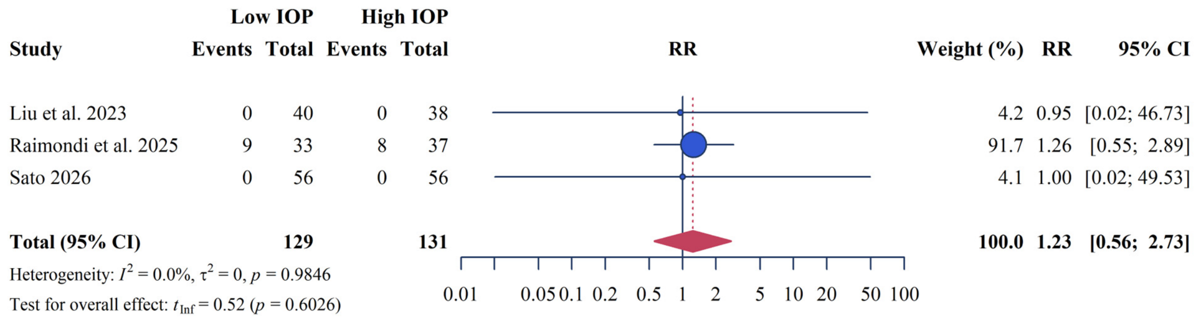

Figure S22. Leave-one-out sensitivity analysis for postoperative corneal edema.

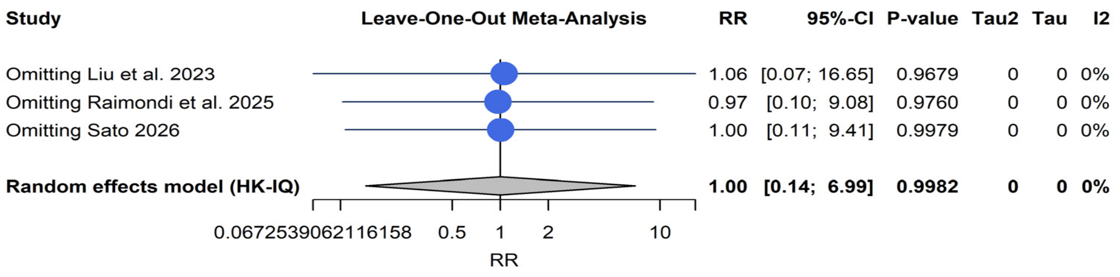

Figure S23. Leave-one-out sensitivity analysis for posterior capsule rupture.

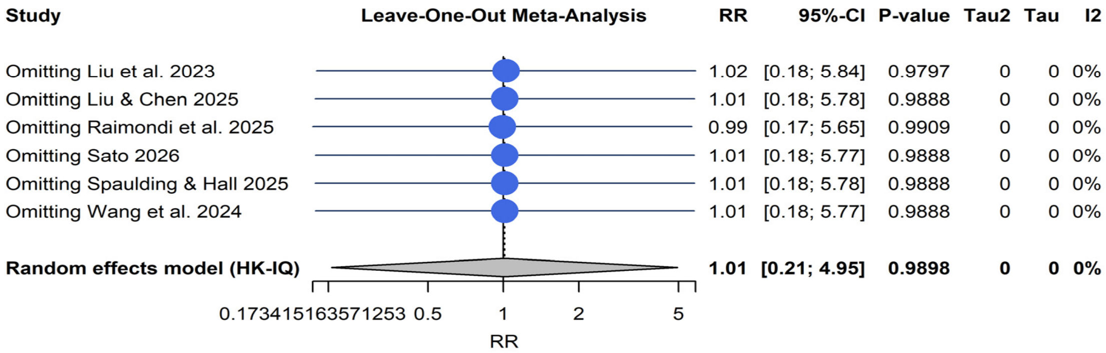

Figure S24. Leave-one-out sensitivity analysis for postoperative IOP elevation.

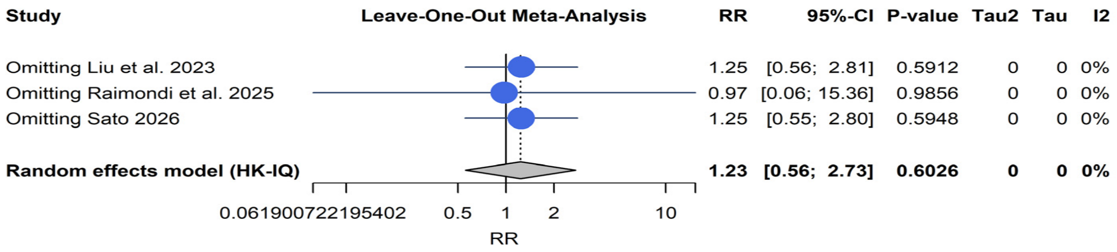

Supplement: Supplementary file 1 [file jcm-15-08557-s001.zip › jcm-4593606-supplementary.pdf]
